# Recombinations, chains and caps: resolving problems with the DCJ-indel model

**DOI:** 10.1186/s13015-024-00253-7

**Published:** 2024-02-27

**Authors:** Leonard Bohnenkämper

**Affiliations:** Faculty of Technology and Center for Biotechnology (CeBiTec), Bielefeld University, Universitätsstraße 25, 33615 Bielefeld, NRW Germany

**Keywords:** Comparative genomics, Genome rearrangement, Double-cut-and-join, Indels, Integer linear programming, Capping

## Abstract

One of the most fundamental problems in genome rearrangement studies is the (genomic) distance problem. It is typically formulated as finding the minimum number of rearrangements under a model that are needed to transform one genome into the other. A powerful multi-chromosomal model is the Double Cut and Join (DCJ) model.While the DCJ model is not able to deal with some situations that occur in practice, like duplicated or lost regions, it was extended over time to handle these cases. First, it was extended to the DCJ-indel model, solving the issue of lost markers. Later ILP-solutions for so called *natural genomes*, in which each genomic region may occur an arbitrary number of times, were developed, enabling in theory to solve the distance problem for any pair of genomes. However, some theoretical and practical issues remained unsolved. On the theoretical side of things, there exist two disparate views of the DCJ-indel model, motivated in the same way, but with different conceptualizations that could not be reconciled so far. On the practical side, while ILP solutions for natural genomes typically perform well on telomere to telomere resolved genomes, they have been shown in recent years to quickly loose performance on genomes with a large number of contigs or linear chromosomes. This has been linked to a particular technique, namely *capping*. Simply put, capping circularizes linear chromosomes by concatenating them during solving time, increasing the solution space of the ILP superexponentially. Recently, we introduced a new conceptualization of the DCJ-indel model within the context of another rearrangement problem. In this manuscript, we will apply this new conceptualization to the distance problem. In doing this, we uncover the relation between the disparate conceptualizations of the DCJ-indel model. We are also able to derive an ILP solution to the distance problem that does not rely on capping. This solution significantly improves upon the performance of previous solutions on genomes with high numbers of contigs while still solving the problem exactly and being competitive in performance otherwise. We demonstrate the performance advantage on simulated genomes as well as showing its practical usefulness in an analysis of 11 *Drosophila* genomes.

**Supplementary Information:**

The online version contains supplementary material available at 10.1186/s13015-024-00253-7.

## Introduction

In genome rearrangement studies, genomes are analyzed on a high level. Most often, the basic unit used is therefore not nucleotides, but oriented genetic *markers*, such as genes. The most fundamental problem in theoretical studies of genome rearrangements is the *distance problem*, which asks to provide the minimum number of rearrangements needed to transform one genome into the other under a restricted set of operations, also called a *model*.

In early approaches, such as the inversion model [1], solutions to the distance problem focused primarily on unichromosomal data, in which each marker appeared exactly once in each genome. These assumptions limited the applications of the models to real biological data, which often contained multiple chromosomes and a wide variety of marker distributions. Since then, researchers have sought to enable models to handle more realistic data. A major breakthrough was the DCJ-model introduced by Yancopoulos et al. in 2005 [2], a simple model that was nonetheless capable of handling multiple chromosomes. In 2010, Braga, Willing and Stoye extended the DCJ-model to the DCJ-indel model, enabling it to handle markers unique to one genome [3]. An independent, equivalent conceptualization of the same DCJ and indel operations was developed by Compeau in 2012 [4], although the precise relationship of the two conceptualizations remained unclear [5]. We refer to these views as the BWS- and Compeau-conceptualization respectively.

In 2021, previous results by Shao et al. [6] were combined with the BWS-conceptualization in [7] to yield the performant ILP solution ding for genome pairs with arbitrary distributions of markers, the so called *natural genomes*. In theory, ding enables the computation of the rearrangement distance between any pair of genomes available today.

However, ding uses a technique known as *capping*, which transforms linear chromosomes into circular ones during solving time. As described in [8], capping increases the solution space of ILPs like ding super-exponentially in the number of linear chromosomes. Since many assemblies available today are not resolved on a chromosome level and instead fragment into sometimes thousands of contigs, this renders distance computation infeasible yet again for many available genomes today. In [8], Rubert and Braga develop a heuristic solution to reduce the search space spanned by capping. Nonetheless, no exact solutions for the DCJ-indel distance problem of natural genomes avoiding capping exist as of yet.

In this work, we apply a new view on the DCJ-indel model developed in [9] to the distance problem. Using this, we are able to bridge the gap between the BWS- and Compeau conceptualizations in  the "Relation of the BWS- and Compeau-Conceptualization" section. Furthermore, this new conceptualization lends itself to a new distance formula (see Theorem 1), which is simple enough to be developed into a capping-free ILP ("Capping-free Generalization to Natural Genomes" section), which we then evaluate in the "Evaluation of the ILP" section to show its performance advantage over ding.

## Problem definition

For this work, we use the same notation as in our previous work. Therefore large parts of this section are adapted from [9]. We conceptualize a genome ${\mathbb {G}}$ as a graph $(X_{{\mathbb {G}}}, M_{{\mathbb {G}}}\cup A_{{\mathbb {G}}})$. Its vertices $X_{{\mathbb {G}}}$ are the beginnings $m^t$ and ends $m^h$ of markers $m:=\{m^t,m^h\}\in M_{{\mathbb {G}}}$. We refer to $m^t,m^h$ as *extremities*. The genome’s * adjacencies* $A_{{\mathbb {G}}}$ are undirected edges $\{m^x,n^y\}\in A_{{\mathbb {G}}}$, which signify that the extremities $m^x$ and $n^y$ are neighboring on the same chromosome. As a shorthand notation, we write *ab* for an adjacency $\{a,b\}$. We require both $A_{{\mathbb {G}}}$ and $M_{{\mathbb {G}}}$ to be a matching on $X_{{\mathbb {G}}}$.

**Fig. 1:**
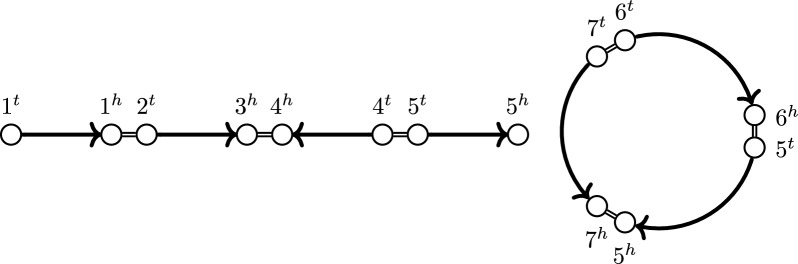
Genome of 7 markers with one linear and one circular chromosome. Markers drawn as arrows, adjacencies drawn as double lines

Because of that requirement, each path in ${\mathbb {G}}$ is simple and alternates between markers and adjacencies. A component of a genome is thus either a linear or circular simple path. We refer to them as linear and circular *chromosomes* respectively. The extremities in which a linear chromosome ends are called *telomeres*. Additionally, we refer to a subpath of a chromosome that starts and ends in a marker a *chromosome segment* (called a *marker path* in [9]). An example of a genome is given in Fig. 1.

In our model, each marker is unique, thus there are no markers shared between genomes. Therefore, in order to calculate a meaningful distance between genomes, we borrow a concept from biology, namely *homology*. Homology can be modeled as an equivalence relation on the markers, i.e. $m\equiv n$ for some $m,n\in M_{{\mathbb {G}}}$. We call the equivalence class [*m*] of a marker *m* its *family*. We also extend the equivalence relation to the extremities with $m^t\equiv n^t$ and $m^h\equiv n^h$ if and only if $m\equiv n$. However, we require that no head is equivalent to any tail, i.e. ${m^t}{\not\equiv} n^h \forall m,n\in M_{{\mathbb {G}}}$. We can then extend the equivalence relation to adjacencies as follows: $ab\equiv cd$ if and only if both of the extremities are equivalent, that is $a\equiv c \wedge b\equiv d$ or $a\equiv d \wedge b \equiv c$.

To illustrate our concept of homology, we introduce the Multi-Relational Diagram (MRD), a graph data structure that is also useful for the distance computation. We deviate from the definition in [7] by omitting indel edges from our definition. This allows us to be closer to the breakpoint graph definition used in [5] and enables the use of the simpler formula in Theorem 1.

### Definition 1

The MRD of two genomes ${\mathbb {A}},{\mathbb {B}}$ and a homology relation ($\equiv$) is a graph ${\mathcal {M}}{\mathcal {R}}D{\mathcal {}}({\mathbb {A}},{\mathbb {B}},\equiv ) = (V,E)$ with $V=X_{{\mathbb {A}}}\cup X_{{\mathbb {B}}}$ and two types of edges $E=E_{\gamma }\cup E_{\xi }$, namely adjacency edges $E_{\gamma }=A_{{\mathbb {A}}} \cup A_{{\mathbb {B}}}$ and extremity edges $E_{\xi }=\{\{x,y\}\in X_{{\mathbb {A}}}\times X_{{\mathbb {B}}} \mid x \equiv y\}$.

**Fig. 2:**
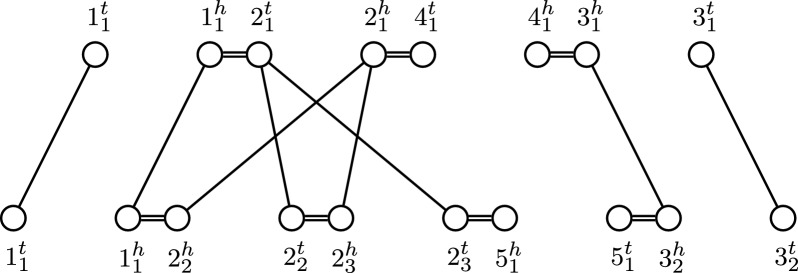
MRD for two genomes on an unresolved homology ($\equiv _1$) with families $\{1_1,1_2\},$ $\{2_1,2_2,2_3\},\{3_1,3_2\},\{4_1\},\{5_1\}$

We give an example of a MRD in Fig. 2. We see that in that example, $4_1$ and $5_1$ have no homologues in the other genome respectively. We refer to such markers as *singular*. Additionally, we call a circular or linear chromosome consisting only of singular markers a circular or linear *singleton*.

Note also that the family $\{2_1,2_2,2_3\}$ in this example has more than just one marker per genome. We call markers of such families *ambiguous*. We refer to a homology, in which no markers are ambiguous as *resolved*. In order to determine the precise nature of rearrangements occurring between two genomes, it is helpful to find a *matching* between the markers of two genomes.

### Definition 2

A *matching* (${\mathop {\equiv }\limits ^{\star }}$) on a given homology ($\equiv$) is a resolved homology for which holds $m{\mathop {\equiv }\limits ^{\star }}n \implies m\equiv n$ for any pair of markers *m*, *n*.

We call two genomes ${\mathbb {A}},{\mathbb {B}}$ *equal* under a homology ($\equiv$), if there is a matching (${\mathop {\equiv }\limits ^{\star }}$) on ($\equiv$), such that each marker and adjacency of ${\mathbb {A}}$ has exactly one equivalent in ${\mathbb {B}}$ under ${\mathop {\equiv }\limits ^{\star }}$ ) and vice versa.

**Fig. 3:**
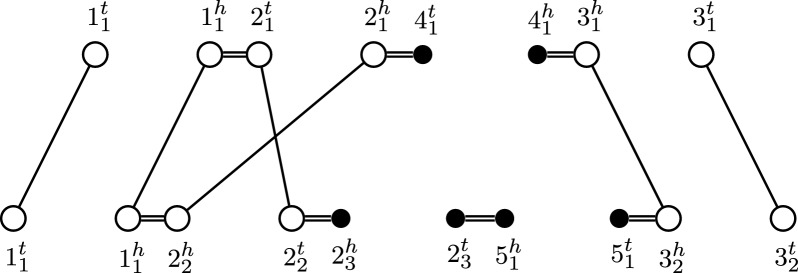
MRD for two genomes on a resolved homology (${\mathop {\equiv }\limits ^{\star }}_1$) with families $\{1_1,1_2\},$ $\{2_1,2_2\}, \{2_3\},\{3_1,3_2\},\{4_1\},\{5_1\}$. Extremities of singular markers (called lava vertices from "A New DCJ-Indel Distance Formula" section onward) are filled black. (${\mathop {\equiv }\limits ^{\star }}_1$) is a (maximal) matching on ($\equiv _1$) of Fig. 2

We note that when the homology is resolved, in the MRD at most one extremity edge connects to each vertex. Because the adjacencies form a matching on the extremities, the resulting MRD consists of only simple cycles and paths. We therefore call such MRDs *simple*. We note that a simple MRD fits the definition of a simple rearrangement graph as studied in Section 3 of [9]. An example of a simple MRD is given in Fig. 3.

Rearrangements in our transformation distance are modeled by the *Double-Cut-And-Join (DCJ)* operation. A DCJ operation applies up to two cuts in the genome and reconnects the incident extremities or telomeres. More formally, we can write as in [10]:

### Definition 3

A *DCJ operation* transforms up to two the adjacencies $ab,cd\in A_{{\mathbb {A}}}$ or telomeres *s*, *t* of genome ${\mathbb {A}}$ in one of the following ways:

- $$ab,cd\rightarrow ac,bd$$ or $$ab,cd\rightarrow ad,bc$$
- $$ab \rightarrow a,b$$
- $$ab,s\rightarrow as,b$$ or $$ab,s\rightarrow bs,a$$
- $$s,t\rightarrow st$$

To model markers being gained or lost, we introduce segmental insertions and deletions.

### Definition 4

An *insertion* of length *k* transforms a genome ${\mathbb {A}}$ into ${\mathbb {A}}'$ by adding a chromosome segment $p=p_{1},p_{2},...,p_{2k-1}p_{2k}$ to the genome. Note that this adds the markers $(p_{1},p_{2}),...,(p_{2k-1},p_{2k})\in M_{{\mathbb {A}}'}$. An insertion may additionally either add the adjacency $p_{2k}p_1\in A_{{\mathbb {A}}'}$, apply the transformation $ab \rightarrow ap_1,p_{2k}b$ for an adjacency *ab* or the transformation $s\rightarrow p_{1}s$ for a telomere *s*. A *deletion* of length *k* removes the chromosome segment $p=p_1,...,p_{2k}$ and creates the adjacency *ab* if previously $ap_1,p_{2k}b\in A_{{\mathbb {A}}}$.

We are now in a position to formulate the distance problem as finding a shortest transformation of DCJ and indel operations of one genome into the other.

### Problem 1

Given two genomes, ${\mathbb {A}},{\mathbb {B}}$ and a homology ($\equiv$), find a shortest sequence $s_1,...,s_k$ of DCJ and indel-operations transforming ${\mathbb {A}}$ into a genome equal to ${\mathbb {B}}$. We call the length of *k* the DCJ-indel distance of ${\mathbb {A}},{\mathbb {B}}$ under ($\equiv$) and write $d_{DCJ}^{id}({\mathbb {A}},{\mathbb {B}}, \equiv ) =k$.

The original DCJ-indel model by Braga et al. [11] only allowed indels on chromosome segments of singular markers to avoid scenarios that deleted and reinserted whole chromosomes. For a resolved homology ${\mathop {\equiv }\limits ^{\star }}$, we call $\overline{d_{DCJ}^{id}} ({\mathbb {A}},{\mathbb {B}},{\mathop {\equiv }\limits ^{\star }})$ the *restricted* DCJ-indel distance if we allow only indels of segments comprised solely of singular markers in scenarios in Problem 1.

For unresolved homologies, we can apply the same model by just finding a matching on the original homology. However, in order to not create a similar “free lunch” issue, we restrict ourselves to an established model, the *Maximum Matching model* [12]. We call a matching (${\mathop {\equiv }\limits ^{+}}$) on a homology ($\equiv$) *maximal* if there are only singular markers in one genome for every family in ($\equiv$).

### Problem 2

Given two genomes, ${\mathbb {A}},{\mathbb {B}}$ and a homology ($\equiv$), find a maximal matching (${\mathop {\equiv }\limits ^{+}}$) on ($\equiv$), such that $\overline{d_{DCJ}^{id}} ({\mathbb {A}},{\mathbb {B}},{\mathop {\equiv }\limits ^{+}})$ is minimized.

## A new DCJ-indel distance formula

We note that the only maximal matching on a resolved homology (${\mathop {\equiv }\limits ^{\star }}$) is (${\mathop {\equiv }\limits ^{\star }}$) itself. Thus, for resolved homologies, in any scenario for Problem 2, we know deletions can only affect singular markers. Let us now regard the MRD of a pair of genomes ${\mathbb {A}},{\mathbb {B}}$ for a resolved homology (${\mathop {\equiv }\limits ^{\star }}$). Since each marker has at most one homologue, each vertex is connected to at most one extremity edge. Since adjacency edges form a matching on the vertices, again, the graph consists only of simple cycles and paths. All cycles are even and we write the set of cycles as $\mathbb {C}_\circ$. Paths can end either in a vertex without an extremity edge or adjacency edges. We name the vertices, in which a path ends in its *endpoints*. Vertices without extremity edges are special, because, as we established earlier, they are the extremities of the markers that will be part of indels during the sorting. We therefore name them *lava vertices*. The other type of vertex are vertices not connected by an adjacency edges. We refer to these as *telomeres*. Note that there is a special case wherein a lava vertex can also be a telomere. We can then identify different types of paths by their endpoints. We write *a* or *b* for a lava vertex and *A* or *B* for a telomere, depending on whether its part of genome ${\mathbb {A}}$ or ${\mathbb {B}}$. We then obtain a partition of paths into 10 different subsets, namely ${\mathbb {P}}_{A\circ A},{\mathbb {P}}_{A|B},{\mathbb {P}}_{B\circ B},{\mathbb {P}}_{A\circ a},{\mathbb {P}}_{A|b},{\mathbb {P}}_{B|a},{\mathbb {P}}_{B\circ b},{\mathbb {P}}_{a\circ a},{\mathbb {P}}_{a|b},{\mathbb {P}}_{b\circ b}$. In order to be consistent with [9], we use $\circ$ and $|$ to distinguish even and odd paths respectively. Furthermore, we write $p_{x(*)y}$ as a shorthand for the cardinality of ${\mathbb {P}}_{x(*)y}$ and $P_{x(*)y}$ for a generic example of an element of ${\mathbb {P}}_{x(*)y}$.

**Fig. 4:**
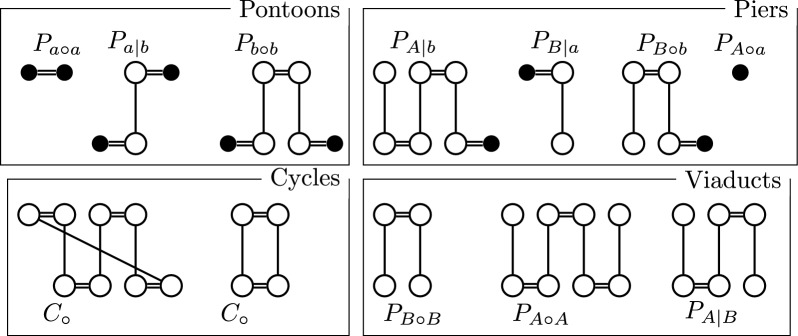
All different types of components in a simple MRD. Vertices of genome ${\mathbb {A}}$ are on the top, vertices of genome ${\mathbb {B}}$ are on the bottom of each component. Lava vertexs are filled black

Usually it is not necessary to think of all 10 different sets as separate entities, because they behave very similarly with respect to applied DCJ or indel operations. In textual form we therefore often use a coarser distinction, naming paths with two lava vertices as *pontoons*, paths with a telomere and a lava vertex as *piers* as well as paths with two telomeres as *viaducts*. An overview of this notation is given in Fig. 4.

**Fig. 5:**
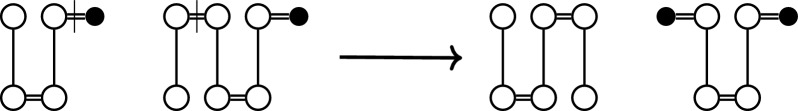
An example of a DCJ operation of the type $P_{A\circ a},P_{B|a}\rightarrow P_{A|B},P_{a\circ a}$

Another notation we adopt from [9] is for a DCJ $ab,cd \rightarrow ac,bd$ affecting the adjacencies *ab* and *cd* in components $K_{ab},K_{cd}$ of the MRD respectively, we can instead view the DCJ as $K_{ab},K_{cd}\rightarrow K_{ac},K_{bd}$ transforming the components $K_{ab},K_{cd}$ into $K_{ac},K_{bd}$. In combination with the generic member notation from above, we can write operations abstractly like so: $P_{A\circ a},P_{B|a} \rightarrow P_{A|B},P_{a\circ a}$. For reference, we have also shown this DCJ operation in Fig. 5.

Based on this notation and with the help of observations from [9], it is possible to derive a new distance formula. We do so in detail in Appendix A. However, this formula is equivalent to that of Compeau and BWS as we will see in the following subsection. We thus only state it here.

### Theorem 1

For two genomes ${\mathbb {A}},{\mathbb {B}}$ and a resolved homology (${\mathop {\equiv }\limits ^{\star }}$) for which both genomes contain no circular singletons, we have the distance formula

$$\begin{aligned} \overline{d_{DCJ}^{id}} ({\mathbb {A}}, {\mathbb {B}}, {\mathop {\equiv }\limits ^{\star }}) = n -c_{\circ } +\bigg \lceil \frac{p_{a |b} + \max (p_{A \circ a},p_{B |a}) +\max (p_{A |b},p_{B \circ b}) - p_{A |B}}{2}\bigg \rceil \end{aligned}$$

with *n* the number of *matched markers*, $n = |\{(m,m')\in M_{{\mathbb {A}}}\times M_{{\mathbb {B}}} \mid m{\mathop {\equiv }\limits ^{\star }}m'\}|$.

Note that constraining ourselves to genomes without circular singletons constitutes no serious restriction, as Compeau showed that circular singletons each require one indel operation and can thus be dealt with in pre-processing [5].

To more easily address individual terms in the formula, we use the followig shorthands,

$$\begin{aligned} F:= n - c_{\circ } + \tilde{P}&:= n- c_{\circ } +\left\lceil {\frac{\tilde{p}}{2}}\right\rceil \\&:= n -c_{\circ } + \left\lceil {\frac{p_{a |b} + \max (p_{A \circ a},p_{B |a}) + \max (p_{A |b},p_{B \circ b}) - p_{A |B}}{2}}\right\rceil . \end{aligned}$$

### Relation of the BWS- and compeau-conceptualization

We now examine how the terms in our distance formula relate to both the Compeau- and BWS-conceptualizations of the DCJ-indel model. In doing that, we uncover the nature of the relation between these two views that have been perceived as entirely separate since their conception [5].

Braga et al. [11] and Compeau [5] use the adjacency and breakpoint graphs respectively. Both graphs are strongly related to the MRD. In fact, one obtains the adjacency graph by collapsing all adjacency edges of a simple MRD and the breakpoint graph by collapsing all its extremity edges. In order to avoid confusion, we will present their results here as if they had been formulated on a simple MRD. When consulting the original works in [5, 11], the reader should keep this in mind. Particularly in [5] the length of a path is determined by its adjacency edges instead of by its extremity edges as defined here. Therefore, parities of viaducts and pontoons are exactly opposite in [5] to the ones stated here.

**Fig. 6:**
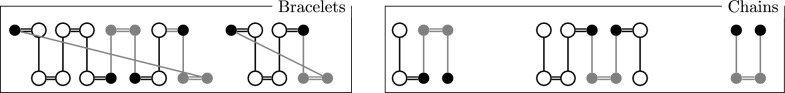
Components resulting from a completion as in [5]. Vertices and Edges added during completion are colored in grey

We will compare the models by examining the chromosome segments that are deleted or inserted (see Definition 4), which we refer to as *indel groups*. We say an adjacency *ab* or its extremities *a*, *b* are *part of an indel group* *p* if there is $a'b'\equiv ab$ with $a'b' \in p$ or *p* starts and ends in $a'$ and $b'$. In terms of indel groups, our view is closely related to the BWS-conceptualization, because both create the indel groups implicitly during sorting (see Appendix A). In terms of the graph, our conceptualization is more closely related to Compeau’s because it essentially operates on the same type of components (lava vertices are called *open* in [5], piers are $\pi$- and $\gamma$-paths and pontoons are $\{\pi ,\pi \}$-, $\{\pi ,\gamma \}$- and $\{\gamma ,\gamma \}$-paths). However, in [5], indels are not modeled as an explicit operation, but instead emulated by integrating or excising artificial circular chromosomes during sorting. Adding the correct chromosomes, the *completion*, is therefore the main problem solved in [5]. These additional chromosomes are then the explicitly constructed indel groups in the sorting. Because the homology of the markers needed for the completion is known beforehand on a resolved homology, the task is to find the correct new adjacencies to add to the graph. Then, if an adjacency $a'b'$ is found in the completion, the extremities $a\equiv a',b\equiv b'$ of the originally singular markers will be part of the same indel group. Once the completion is constructed, there are no more lava vertices in the graph. Instead, former piers and pontoons are joined into new components, either *bracelets*, which are circular and consist of pontoons only, or *chains*, which consist of two piers and possibly pontoons. An example of a completion can be found in Fig. 6.

**Fig. 7:**
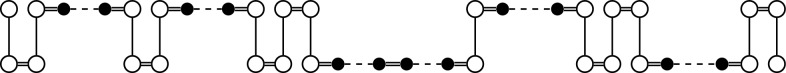
Path as found in [7] as another way of writing the paths in [11] by adding indel edges between lava vertices of the same gene. Indel edges here drawn in dashed. In this work, indel edges are omitted and the collection of components arising is called a *bridge*

In [11], lava vertices are avoided by viewing singular markers as part of adjacencies of matched markers, called $\mathcal {G}$-*adjacencies*. This is equivalent to connecting the head and tail vertex of a singular marker with a special type of edge, called *indel edge* as is done in [7]. Introducing indel edges concatenates components with lava vertices. We name these concatenated component *crossings* and distinguish between circular crossings called *ferries* and linear crossings called *bridges*.

#### Definition 5

A *pontoon bridge* $b_1,..,b_k$ for $k\ge 2$ is a string of components $b_i$, such that $b_1,b_k$ are piers, $(b_i)_{i=2}^{k-1}$ are pontoons and there are singular markers $(m_i)_{i=1}^{k-1}$ with $m_i\ne m_j$ for $i\ne j$ whose extremities are contained as lava vertex in $b_i,b_{i+1}$ for all $m_i$. A string of components is called a *bridge* if it is a pontoon bridge or consists of a single viaduct.

#### Definition 6

A *pontoon ferry* $f_1,...,f_l$ for $l\ge 1$ is a string of pontoons $f_i$, such that here are singular markers $(m_i)_{i=1}^{l}$ with $m_i\ne m_j$ for $i\ne j$ whose extremities are contained as lava vertices in $f_i,f_{i+1}$ for all $m_i$ for $i<l$ and the extremities of $m_l$ are contained in $f_1$ and $f_l$. A string of components is called a *ferry* if it is a pontoon ferry or consists of a single cycle.

Ferries and bridges are cycles and paths in [11] respectively. An example of a bridge can be found in Fig. 7. Crossings are first sorted separately in [11], so we start our comparison by doing the same. We thus aim to find *internal* operations that only involve components of the same crossing. During sorting, we want to make sure that the operations we apply are not only optimal in the context of the crossing, but in the graph as a whole. There are certain operations that are guaranteed to be optimal because they reduce $F$ in any MRD by 1, no matter which other components are found in the graph. We call such an operation *safe*. For example, extracting a cycle from any component is safe (as $\Delta c_\circ = 1$), whereas recombining two even piers, such as $P_{A\circ a},P_{A\circ a} \rightarrow P_{A\circ A},P_{a\circ a}$ is not safe, because it is only optimal under the premise that $p_{A\circ a} > p_{B|a}$. There are only 7 distinct types of safe DCJ operations. We list them in Table 1. We also note that as in [11], instead of sorting ${\mathbb {A}}$ to ${\mathbb {B}}$, we can sort both ${\mathbb {A}}$ and ${\mathbb {B}}$ to a common genome. By thinking this way, we can better exploit the symmetry of the situation.

**Table 1:** All safe types of DCJ operations

Safe operation	$-\Delta c_\circ$	$\Delta p_{a|b}$	$\Delta \max (p_{A\circ a},p_{B|a})$	$\Delta \max (p_{A|b},p_{B\circ b})$	$-\Delta p_{A|B}$
$K\rightarrow K'+C_\circ$	− 1	0	0	0	0
$P_{A\circ A}\rightarrow P_{A|B},P_{A|B}$	0	0	0	0	− 2
$P_{B\circ B}\rightarrow P_{A|B},P_{A|B}$	0	0	0	0	− 2
$P_{A\circ A},P_{B\circ B}\rightarrow P_{A|B},P_{A|B}$	0	0	0	0	− 2
$P_{a|b},P_{a|b}\rightarrow (P_{a\circ a})^*,(P_{b\circ b})^*$	0	− 2	0	0	0
$P_{A\circ a},P_{B|a} \rightarrow P_{A|B},(P_{a\circ a})^*$	0	0	− 1	0	− 1
$P_{A|b},P_{B\circ b}\rightarrow P_{A|B},(P_{b\circ b})^*$	0	0	0	− 1	− 1

Each reduces the $F$ by 1, no matter the number of other components in the graph. Above are all safe operations in a pure DCJ scenario. The operations below can also function as safe deletions if one of the resultants in brackets is removed. For reference: $F= n -c_{\circ } + \left\lceil {(p_{a |b} + \max (p_{A \circ a},p_{B |a}) + \max (p_{A |b},p_{B \circ b}) - p_{A |B})/2}\right\rceil$

**Fig. 8:**
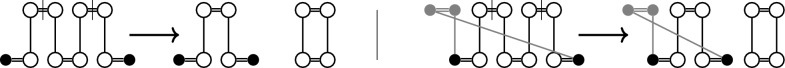
Safe DCJ operations accumulating markers separated by even pontoons (in [11] called a *run*) remain optimal in the safe bracelet joining the extremities of these markers

The most obvious safe operation is the extraction of an even cycle from another component. If one continues to extract even cycles from an even pontoon $p=x_1...x_k$ with lava vertices $x_1$ and $x_k$, one arrives at the pontoon $p'=x_1x_k$, which consists of a single adjacency. The corresponding singular markers of $x_1$ and $x_k$ can then be dealt with with the same indel operation, meaning $x_1,x_k$ are part of the same indel group. Braga et al. notice the same thing in [11]; they refer to markers that are only separated by even pontoons as a *run*, which they notice can be “accumulated” in this fashion. For an extensive example, see Additional file 1: Fig. S16 in Appendix B, Steps (a), (b). In [5], genomes are not explicitly sorted, so there is no true equivalent to safe operations, but Compeau systematically finds chains and bracelets he can be sure are optimal in any breakpoint graph (Algorithm 9, Steps 1 to 3). We therefore call these chains and bracelets safe, too. In fact, the very first safe bracelet Compeau identifies, is a 1-bracelet consisting of a single even pontoon (Lemma 5 in [5]). If one creates this bracelet from the even pontoon $p=x_1...x_k$ the adjacency added for the completion is $x_1'x_k'$ with $x_1\equiv x_1'$ and $x_k\equiv x_k'$. Thus, here too, $x_1x_k$ are part of the same indel group. This way of constructing the indel groups is shown in Additional file 1: Appendix B, Fig. S17 with Bracelets (a), (b). Notice also that the safe operations sorting the two adjacent lava vertices of an even pontoon together remain optimal in a bracelet like this (see Fig. 8).

**Fig. 9:**
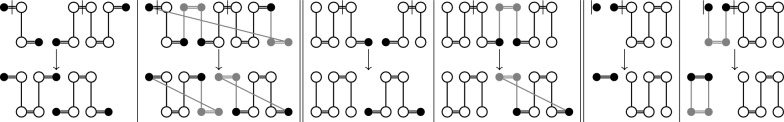
For all safe DCJ operations with two piers or pontoons as sources, there is a safe bracelet or chain in which the same operation is optimal and vice versa

The next safe bracelet Compeau finds, is joining two odd pontoons together. He shows that it is safe by ruling out all other uses of two pontoons as at best co-optimal (Lemma 6, Proof of Thm 8 and Step 2 of Algorithm 9 in [5]). An example can be found as Bracelet (c) of Additional file 1: Fig. S17. This again, corresponds to a safe operation, namely $P_{a|b},P_{a|b}\rightarrow P_{a\circ a},P_{b\circ b}$. In fact, all safe chains and bracelets of two components correspond directly to safe operations. We have visualized this fact in Fig. 9. Note that the corresponding safe operation again remains optimal in the safe chain or bracelet. Because of this more direct correspondence between the Compeau-conceptualization and our formula, we focus more on the correspondence between the BWS-conceptualization and our formula in the following. Braga et al. identify the same operation by noticing that the number of runs can be reduced by 2 if one applies cuts in between between runs of ${\mathbb {A}}$ and ${\mathbb {B}}$ (see Proposition 3 in [11]). This is of course precisely a DCJ with two odd pontoons as sources in our model. Because the resultants of this operation are the two even pontoons $P_{a\circ a},P_{b\circ b}$, these can in turn be reduced to single adjacencies by excising even cycles. Again, the implication for indel groups in all models is that for two odd pontoons $p_1=a_1x_1,...,x_kb_1,p_2=a_2x_{k+1},...,x_lb_2$, the adjacency $a_1a_2$ can be part of the same indel group if $b_1b_2$ is part of the same indel group and vice versa. This equivalence is further illustrated by comparing the effects of Steps (c) and (d) of Additional file 1: Fig. S16 to Bracelet (c) of Additional file 1: Fig. S17 of Appendix B.

Dealing in this fashion with all pontoons of a crossing, we reduce all but possibly one odd pontoon to single adjacency edges, which can then be dealt with in a single indel operation. Because ferries must contain an even number of odd pontoons, they can be sorted entirely by safe operations in this way. To quantify the number of operations needed, Braga et al. define the *indel potential* $\lambda (X)$ of a crossing *X* as the number of indel operations obtained in a DCJ-optimal sorting [11]. Since it is possible to trade off indel and DCJ operations, this definition is not easily reflected in the other conceptualizations. However, as they show that sorting a crossing *X* separately needs $d_{DCJ}^{id}(X)=\textrm{d}_{DCJ} (X) + \lambda (X)$ steps, we can also think of the indel potential as the overhead introduced by the singular markers if we sort the crossing separately. In [11], it is shown that $\lambda (X)=\left\lceil {\frac{\Lambda (X)+1}{2}}\right\rceil$ with $\Lambda (X)$ the number of runs for a crossing *X*. If a ferry contains at least two runs, we can find a bijection between runs and odd pontoons. Denoting $q(X)$ as the contribution to quantity *q* by crossing *X*. We can thus write $\Lambda (X) = p_{a|b}(X)$ for a ferry with at least two runs. Therefore, we find for a ferry *X* with at least two runs, their formula translates to ours,

$$\begin{aligned} n(X)- c(X) +\lambda (X)&= n(X) -c(X) +\left\lceil {\frac{\Lambda (X)+1}{2}}\right\rceil \\&= n(X)-1 + \frac{\Lambda (X) +2}{2} = n(X) + \frac{\Lambda (X)}{2} = n(X) +\left\lceil {\frac{p_{a|b}(X)}{2}}\right\rceil . \end{aligned}$$

Similarly, this equivalence can be shown if there is only 1 run in *X*. By the Compeau method, if there are *d* singular markers, *d* markers are added as part of completion chromosomes, so the number of markers after completion is $N=n+d$. Meanwhile, each $P_{a\circ a}$ and $P_{b\circ b}$ creates a bracelet. Each pair $P_{a|b},P_{a|b}$ also forms a bracelet. Since $d=p_{a|b} + p_{a\circ a}+p_{b\circ b}$, we have

$$\begin{aligned} n(X) + \left\lceil {\frac{p_{a|b}(X)}{2}}\right\rceil &=n(X) + \frac{p_{a|b}(X)}{2} = n(X) -\frac{p_{a|b}(X)}{2} + p_{a|b}(X)\\&=n(X) + d(X) - \frac{p_{a|b}(X)}{2} -p_{a\circ a}(X) - p_{b\circ b}(X) \\&= N(X) - \left( p^{\pi ,\pi }(X)+p^{\gamma ,\gamma }(X) +\left\lfloor {\frac{p^{\pi ,\gamma }(X)}{2}}\right\rfloor \right) , \end{aligned}$$

which is precisely the Compeau formula if no piers or viaducts are involved. We see that our formula acts as a sort of missing link between the two other formulas here. Since ferries can be dealt with entirely with internal safe operations, this formula can even be generalized to the whole graph for circular genomes. In fact, this has been done in [7], yielding our formula for this specific case.

Using this way of examining the contribution of individual crossings, we were also able to re-calculate the indel potential with our formula for all 10 types of bridges in [11]. The results can be found in Additional file 1: Table S4 of Appendix B. Notably, when sorting a bridge independently, one can also first exhaust all safe operations. After this, only the piers and possibly a single odd pontoon might be “left over” (see also Additional file 1: Fig. S16 after Step (d)). We call these components *unsaturated*. Since each safe operation also has a corresponding safe chain or bracelet, these are also the only components, which end up in unsafe chains if one restricts the completion to a single crossing (compare to Additional file 1: Fig. S17). Since every other component can be dealt with safe operations, unsaturated components are the only ones that might have to be involved in what is called in [11] a *(path) recombination*, that is, a DCJ operation involving more than one crossing. When studying recombinations, we can therefore abstract from any concrete bridge $p=p_1,...,p_k$ with piers $p_1,p_k$ and only write it as its unsaturated components. We therefore write such a component as $p_1p_k$ if *p* contains an even number of odd pontoons or as $p_1P_{a|b}p_k$ otherwise. We call this the *reduced bridge*. Interestingly, Braga et al. make the same abstraction and identify the bridges by the genome of their telomeres and the genome of the first and last run. This direct correspondence is illustrated by comparing Columns 1 and 4 of Additional file 1: Table S4. In [11], another observation is that (reduced) bridges of the type $P_{A|b},P_{B\circ b}$ or $P_{A\circ a},P_{B|a}$ never need to appear as sources for any recombination. Using our conceptualization, we can confirm that because $P_{A|b},P_{B\circ b}\rightarrow P_{A|B},P_{b\circ b}$ and $P_{A\circ a},P_{B|a}\rightarrow P_{A|B},P_{a\circ a}$ are safe operations, these types of bridges can be sorted entirely by internal safe operations. It therefore makes sense to group them as in [11] with viaducts, the other type of bridge that can be sorted in this way.

All other bridges might need recombinations to be sorted optimally. If there is a safe operation between the components of two bridges, we know that this recombination must be optimal. In fact, if we only regard unsaturated components, we see that the only remaining safe operations are (i) $P_{A\circ a},P_{B|a}\rightarrow P_{A|B},P_{a\circ a}$, (ii) $P_{A|b},P_{B\circ b}\rightarrow P_{A|B},P_{b\circ b}$ and (iii) $P_{a|b},P_{a|b}\rightarrow P_{a\circ a},P_{b\circ b}$. We know (either by combinatorics or Additional file 1: Table S4) that each source of (i) and (ii) appears in 3 types of (reduced) bridges and thus there are $3\times 3=9$ path recombinations facilitated by each of these two safe operations. For (iii), we have 4 types of (reduced) bridges containing $P_{a|b}$ and thus $$\left( {\begin{array}{c}4\\ 1\end{array}}\right) +\left( {\begin{array}{c}4\\ 2\end{array}}\right) =10$$ path recombinations using this operation. Of course, these are not mutually exclusive, but since Operations (i) and (ii) involve the end of a bridge and one of its resultants is a viaduct, we can always choose to do one of these operations first, upon which all other possible safe operations on piers and pontoons will be in the same component and will not require any further recombinations. In [11] all of these recombinations are catalogued. We were able to confirm this by recreating their tables of recombinations with $\Delta d \le 0$ as Additional file 1: Table S5 of Appendix B. It is easily checked that (i) and (ii) each occur 9 and (iii) occurs 10 times. The unsaturated components after the operation in these cases form precisely those bridges listed in [11] as the resultant(s). The precise difference for the distance as opposed to sorting the crossing separately can then be derived by comparing the term $\tilde{P}$ in our formula on the graphs containing each bridge separately and on a graph containing the union of the two bridges (see Additional file 1: Table S5 Columns 3, 6, 9, 10). In summary, we can see that in all but two cases, the DCJ chosen to recombine the bridges in [11] is safe and the resultants are exactly comprised of the unsaturated components after the operation.

The two exceptions are the recombinations of $P_{A\circ a},P_{A\circ a}$ with $P_{B\circ b},P_{B\circ b}$ and $P_{A|a},P_{A|b}$ with $P_{B|a},P_{B|a}$ (marked with $\star$ in the table). In these cases, there is no safe operation and therefore all piers remain unsaturated. The reason this recombination can still be done in some cases is that an unsafe operation like $P_{A\circ a},P_{B\circ b}\rightarrow P_{A|B},P_{a|b}$ in this specific case reduces $F$ by one, but since there are equally optimal internal operations (i.e. $P_{A\circ a},P_{A\circ a}\rightarrow P_{A\circ A},P_{a\circ a}$) in this case, this recombination actually never has to be used. The only task remaining is then to find a sequence of recombinations that improve upon the distance. Braga et al. give this as their *recombination groups*. We have listed these groups in Additional file 1: Table S6 of Appendix B. The first observation is that by exhausting all safe DCJ operations in a recombination group, we are able to create the unsaturated components of what are called in [11] *reusable resultants*. In combination with our observations about pairwise recombinations, we thus know that all recombinations in the groups can be facilitated purely by safe DCJs. We also see that in many cases, after sorting a group, no further unsaturated components are present. In the other cases, Braga et al. make sure that all partners of the unsaturated components are “used up” in earlier recombinations of the table (see last column) or that remaining safe operations are at most co-optimal, such that the unsafe operations sorting the unsaturated components are still optimal.

## Capping-free generalization to natural genomes

**Algorithm 1:**
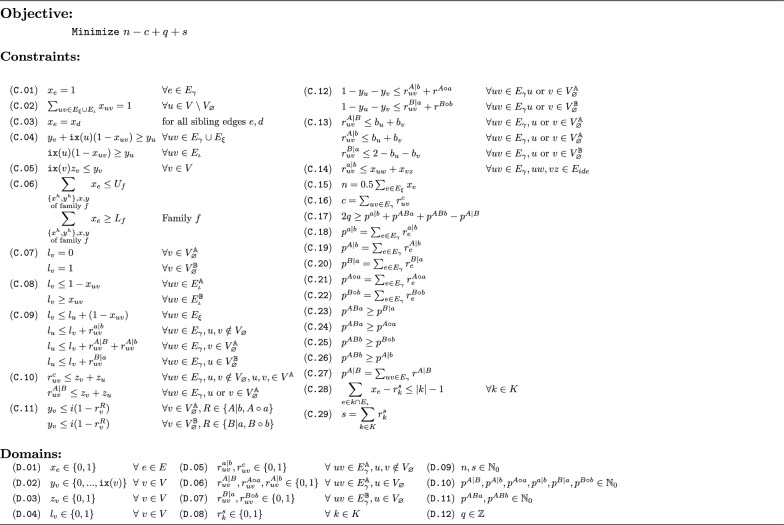
Capping-free ILP to compute the DCJ-indel distance for natural genomes

**Fig. 10:**
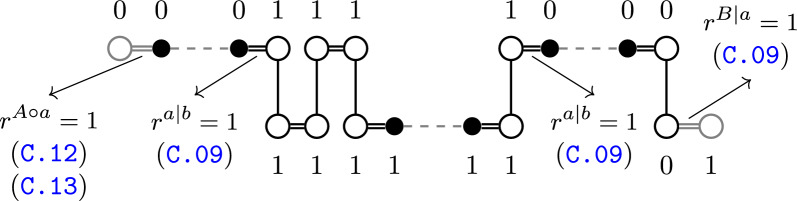
Modifications to the MRD for the ILP: Pseudo-caps and additional adjacency edges shown in grey, indel edges shown dashed. Numbers show the optimal variable assignment of the *l*-variable for each vertex. Arrows indicate the setting of a report variable of an edge together with the constraints responsible. Other report variables are 0. Due to C.04 all *y*-variables are 0 and consequently (C.05) all *z* variables are 0.

In this section, we describe briefly how to generalize the distance formula presented as Theorem 1 to an ILP for which no capping of the MRD is required. The ILP works by determining a matching on the markers as described in Problem 2. Equivalent to finding a matching is to find a *decomposition* of the MRD, that is, a subset of extremity edges, such that each vertex is connected to at most one extremity edge. In this case, it is important that the decomposition is *consistent*, meaning the matched extremity edges should express the same matching of the markers. More formally, if $m\in M_{{\mathbb {A}}}, n \in M_{{\mathbb {B}}}$ with $m\equiv n$ then the extremity edge $n^tm^t$ is part of a consistent decomposition if and only if $n^hm^h$ is. We call the edges $n^tm^t$ and $n^hm^h$ *siblings* [7].

Before we give the ILP, we describe the modifications to the MRD necessary to construct the program.

Firstly, in order to be consistent with predecessor ILPs, we retain indel edges as used in [7]. Indel edges connect the head and tail vertices of a marker and we denote their set by $E_{\iota }$. However, we do not use them to connect form bridges and ferries as in the previous section. Instead, we still use the distinction of components and formula of Fig. 4. We use an indel edge merely to indicate when a marker’s vertices are not connected to any extremity edges in the decomposition and thus the vertices are lava vertices.

To avoid the edge case of a path with only a single vertex and no edges, we apply a slight modification for telomeres: For each telomere *v*, we add another vertex ${v}_{\varnothing }$ and add $v{v}_{\varnothing }$ as an adjacency edge. We name these added vertices *pseudo-caps* and write the set of these vertices as $V_{\varnothing }$. An example of these modifications can be found in Fig. 10.

Note that in contrast to “real” capping as applied in [7], pseudo-caps do not significantly increase the solution space, because they are not connected by extremity edges, which would need to be resolved as part of finding a decomposition.

Finally, each vertex *v* of the MRD is assigned a unique identifier $\texttt {ix}(v)$ with $\texttt {ix}(v) \ge 1$. We assign vertices of genome ${\mathbb {A}}$ lower identifiers than vertices of genome ${\mathbb {B}}$. Since we continue to make some distinctions based on the genome vertices or edges in, we use the notation $S^{\mathbb {A}}$ and $S^{\mathbb {B}}$ to stand for the subsets of a set *S* with elements in genome ${\mathbb {A}}$ and ${\mathbb {B}}$ respectively. For reasons that will become clear later, we assign pseudo-caps the lowest ids, that is, $\forall v \in V_{\varnothing }^{\mathbb {A}}, \forall u \in V_{\varnothing }^{\mathbb {B}}, \forall w \in V:\, \texttt {ix}(v)<\texttt {ix}(u) <\texttt {ix}(w)$.

We now begin the description of the ILP. The basic framework to compute consistent decompositions (Constraints C.01 to C.06) is the same as for ding [7] and the ILP by Shao et al. [6]: A binary variable *x* is used to indicate whether or not an edge is part of the decomposition. Variable $y_v$ in an optimal solution is equal to the lowest vertex id of in the component and $z_v$ marks the vertex *v* with the lowest index $\texttt {ix}(v)$ in a component without lava vertices. In components with lava vertices, all *y*-variables and consequently all *z*-variables are set to 0. We also adopt the way circular singletons are dealt with in [7] as Constraint C.28.

The only major change we make w.r.t. [7] in Constraints C.01 to C.06 is the addition of Constraint C.06, where we allow for other matching models in addition to the maximum matching model by specifying an upper ($U_f$) and lower bound ($L_f$) for the number of markers to be matched per family *f*. Specifications for how to set these bounds to achieve the maximum matching model and other popular models can be found in Table 2.

Our goal is to find the consistent decompositions with the lowest DCJ-indel distance (see Problem 2). To calculate the DCJ-indel distance for the objective, we then need to distinguish the different components from each other. We thus have to distinguish the 11 types of cycles, viaducts, piers and pontoons from each other (see Fig. 4 for a reminder of what these components look like).

From a birds-eye view, we detect the type of a component via binary report variables anchored at adjacency edges. These are named named $r^C_e$ for reporting component type *C* at edge *e* (see Domains D.05 to D.08). We then sum up these report variables to obtain the terms of the formula (see C.16 to C.27) with variable *q* representing the fraction $\tilde{P}$. Together with *n*, the number of markers in the decomposition (C.15) and *s*, number of circular singletons (C.29), we are able to construct our distance formula for the objective function.

Of course, we need to ensure that an $r^C$-variable is set to 1 only once per component as well as if and only if the component type is actually *C*. To detect, in which genome the endpoints of a path lie, we use the label variable $l$ (D.04). This variable is set to 0 for endpoints in genome ${\mathbb {A}}$ and to 1 for endpoints in genome ${\mathbb {B}}$. This is done statically for pseudo-caps (C.07) and dynamically for other vertices if they become lava vertices (C.08). We require this variable to be the same for two connected vertices (C.09), but escape the cases when an edge is not part of the decomposition ($1-x_{uv}$) or when reporting a component type with endpoints in both genomes ($r^{a|b},r^{A|B},r^{A|b},r^{B|a}$).

Because telomeres are known beforehand and marked by pseudo-caps, we can make sure one telomere endpoint of the path an *r* variable reports is correct by only defining the corresponding *r* variable on adjacency edges involving a pseudo-cap in the correct genome (see Domains D.06, D.07).

For reporting cycles, and viaducts of type $P^{A|B}$, we require the *z* variable of an adjacent vertex to be set to 1 (C.10). This serves two purposes: On the one hand, it ensures that *r* is only set to 1 once per component (as there is only one *z* variable set to 1 per component). On the other hand, it ensures that no component containing lava vertices can report $P^{A|B}$ or a cycle. This is because components with lava vertices do not have any *z*-variables set to 1. This is important because cycles as well as viaducts of type $P^{A|B}$ decrease the formula while all other component types are either neutral or increase the formula.

Conversely, we set all *y*-variables and by proxy all *z*-variables to 0 if a pier is reported (Constraint C.11). This is to prevent the report variable being used to alter the $l$-variable of $P^{A\circ A}$ (or $p^{B\circ B}$) type paths to report a $P^{A|B}$ path.

With the constraints described until now, the ILP would only correctly report piers with endpoints in different genomes, because while Constraint C.09enforces that any $P^{A|b}$ or $P^{B|a}$ are reported, there is no equivalent for the type $P^{A\circ a}$ or $P^{B\circ b}$. We therefore require for pseudo-caps to report a pier if the *y*-variable is 0 (C.12), which indicates that there is a lava vertex in the component due to Constraint C.04. The interplay of *l*, the *r* variables and selected constraints is also visualized in Fig. 10.

Additionally, we require a change in the $l$-variable when reporting odd paths, such that no even path can be reported as an odd one ( C.13).

To enforce that $r^{a|b}$ is set only in components with lava vertices, we use an idea introduced in [7]: We require for $r^{a|b}$ to be set to 1 that a neighboring indel edge is part of the decomposition ( C.14).

Note here that we do not prevent the variable $r^{a|b}$ to be used to change $l$ in components containing lava vertices as we did for other report variables before. For example, it is possible that in a component $P^{A\circ a}$ any $r^{a|b}$ variable could be set to 1, meaning $r^{A|b}$ could be set to 1 at the pseudo-cap instead of $r^{A\circ a}$. However, since any $r^{a|b}$ variable set to 1 increases the formula at least as much as the report variable of any pier type, this has no effect on optimal solutions.

**Table 2:** Settings for $$U_f,L_f$$ in Algorithm 1 to enforce different matching models described in [12] with $f_\mathbb{A}$ and $f_\mathbb{B}$ the markers of $f$ in $\mathbb{A}$ and $\mathbb{B}$ respectively

	Maximum matching (Full)	Intermediate matching	Exemplar matching
$L_f$	$\min (|f_\mathbb{A}|,|f_\mathbb{B}|)$	$\min (|f_\mathbb{A}|,|f_\mathbb{B}|)$	1
$U_f$	$\min (|f_\mathbb{A}|,|f_\mathbb{B}|)$	1	1

## Evaluation of the ILP

We implemented the ILP described in the previous section and made it publicly available.^1^ We refer to this implementation as ding-cf for the rest of this work.

In this section, we show results of applying the ILP to both simulated and real data and comparing its performance to the python3 version of ding [7], namely dingII, a similar ILP solution to the DCJ-indel distance problem for natural genomes. In contrast to ding-cf, dingII uses the capping technique. In all experiments, we used gurobi10.0 on a single thread on a virtual machine with 256 GB RAM to solve the ILPs.

We first test the ILPs on simulated data in  "Performance Evaluation on Simulated Data" Subsection before demonstrating the practical usefulness of rearrangement analyses on contig level resolved genomes by analysing 11 Drosophila genomes in "Analysis of Drosophila Genomes" Subsection.

### Performance evaluation on simulated data

We initially planned to use the simulation script that comes with dingII, but due to the script regularly encountering stack overflows on large genomes owing to its reliance on recursion, we instead re-implemented it in C++. This implementation is also publicly available.^2^

The re-implementation has the same features as the original script with only one minor change: Instead of the number of DCJ-operations to be performed as a parameter, our simulation takes a fixed number of total operations and distributes them according to rates relative to a rate of 1 for DCJ operations. For more detail on the simulation, the interested reader is referred to the description of the original simulation script in [7].

In our experiments, we simulated two genomes from a common root for each sample. We chose parameters close to those of the experiments performed in the original ding publication [7]. In all experiments, we set the length of the root genomes to 20,000 markers and performed 10,000 operations in total, with an insertion rate of 0.1 and an deletion rate of 0.2 unless specified otherwise. For reference, this amounts to 5882 DCJ operations in expectation for a duplication rate of 0.4 to compare to experiments run with the python script of dingII. The shape parameter for the Zipf distribution was set to 4 for indel lengths and to 6 for duplication lengths. In the experiments of this section, we limited gurobi’s solving time to 1*h* (3600*s*). All experiments were designed to test parameters to which ILPs like ding have been shown to be sensitive.

**Fig. 11:**
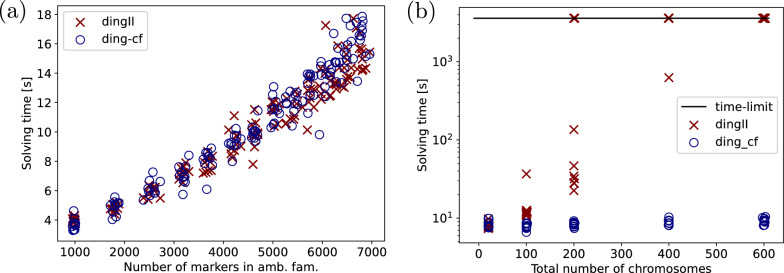
Runtimes for dingII and ding-cf for genomes simulated in 10,000 steps from a common root, in **a** increasing the duplication rate in steps of 0.1 from 0.1 to 1.1, in **b** increasing the number of linear chromosomes in the root genome progressively from 10 to 50 to 100, 200 and 300

In our first experiment, we increased the duplication rate in steps of 0.1 from 0.1 to 1.4, generating 10 genome pairs from a root genome with 1 linear chromosome per step. We then created the ILPs for dingII and ding-cf. The number of ambiguous families ranged from 628 to 4356 (median 3076) in this experiment with the maximum family size per sample reaching up to 7 markers.

We show the solving times of gurobi10.0 in Fig. 11 (a). We see that ding-cf is competitive with dingII. This is not surprising as most additional constraints of ding-cf w.r.t. dingII are due to the pseudo-caps and thus do not overwhelmingly come into effect as long as the number of linear chromosomes is low. We were able to further verify that on genomes with few linear chromosomes, ding-cf behaves similarly to ding for varying different parameters in Additional file 1: Appendix C.

**Fig. 12:**
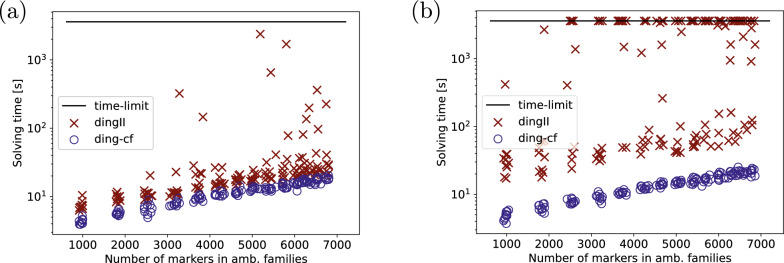
Runtimes for dingII and ding-cf for genomes simulated in 10,000 steps from a common root increasing the duplication rate in steps of 0.1 from 0.1 to 1.1 with **a** 100 total linear chromosomes and **b** 200 total linear chromosomes on average per sample pair

To test the actual use case for ding-cf, that is, high numbers of linear chromosomes, we increased the number of linear chromosomes in the root genome progressively from 10 to 50 to 100, 200 and 300 chromosomes with a fixed duplication rate of 0.4 and 10 samples per step. The runtimes are shown in Fig. 11 (b). We see that up to 100 linear chromosomes in the simulated pair of genomes, dingII is able to compete with ding-cf, but its runtime rises exponentially until the majority of the dingII ILPs are not solved within an hour of solving time. Meanwhile, the runtimes of ding-cf are stable throughout the experiments, staying below 20 seconds in each case.

In order to test the composite effect of the number of duplicates and the number of linear chromosomes on solving times, we repeated the first experiment (Fig. 11 (a)) with 50 and 100 linear chromosomes at the root genome, resulting in total numbers of about 100 and 200 linear chromosomes for each pair. The results (shown in Fig. 12) indicate that dingII is far more sensitive to changes in the number of chromosomes with the first increase to 100 chromosomes in the pair already showing longer solving times on most samples. The second increase to 200 fully separates the two solutions, having a negligible effect on ding-cf while making the solving times for dingII much more unpredictable. Many of the pairs with high duplicate numbers become unsolvable within an hour for dingII.

**Fig. 13:**
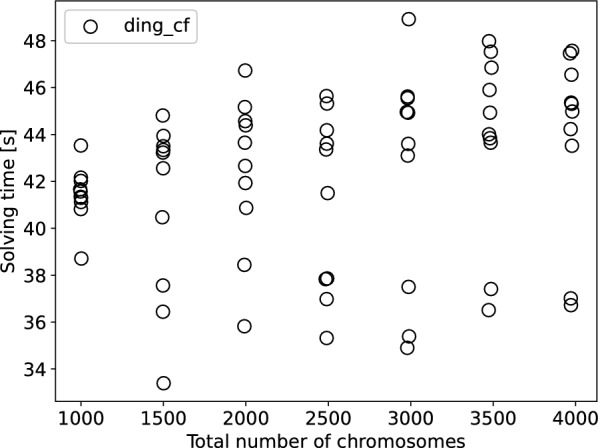
Runtimes for ding-cf for genomes simulated from a root with 500 to 2000 chromosomes in steps of 250

To confirm that the number of linear chromosomes alone only plays a minor part in the runtime of ding-cf, we ran another experiment, this time keeping the duplication rate fixed at 0.4 and increasing the number of linear chromosomes in the root genome from 500 to 2000 in steps of 250 with 10 samples per step. The runtimes are given in Fig. 13 and exhibit only a minor, linear increase. In fact, the increase is so slow that even for 2000 linear chromosomes at the root (c.a. 4000 linear chromosomes of the pair in total), the runtime is still below a minute for all 10 samples.

### Analysis of drosophila genomes

We obtained 11 assemblies of species in the *Drosophila* genus previously analyzed by Rubert and Braga [8]. We used FFGC to extract the longest transcript of each locus and ran OrthoFinder version 2.3.7 [13] to obtain orthologous groups. We then translated the genomes into unimog files using the orthogroups as families and translating linear contigs into linear chromosomes. We then filtered out any empty chromosomes. The genomes obtained in this fashion comprised 13,143 markers spread on 97 linear chromosomes on average. More detailed statistics about the genomes after this preprocessing step are listed in Additional file 1: Table S7 of Appendix D.

We then used ding-cf to calculate pairwise distances, running gurobi10.0 on a single thread for 12 h. Of the 55 resulting ILPs, we obtained an exact result for 9 and approximate results for 46, all of which deviated at less than 2% from the exact solution, most of them below 1%. We give the distance data obtained in this manner and detailed performance results in Additional file 1: Table S8 of Appendix D.

**Fig. 14:**
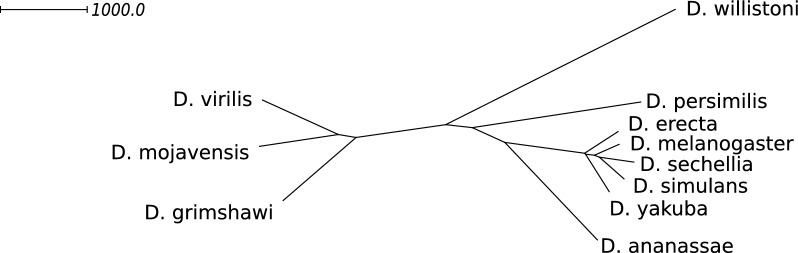
Neighbor joining tree inferred from the distances in Additional file 1: Table S8 using SplitsTree4. Edge lengths are drawn proportional to their weight. The absolute edge lengths can be found in Additional file 1: Appendix D

*Phylogenetic Analysis.* We proceeded to construct a phylogenetic tree via Neighbor Joining using SplitsTree4 [14]. The tree, shown in Fig. 14, is entirely consistent with the current state of knowledge about the Drosophila phylogeny. Additionally, the phylogenetic signal in the distance data is remarkably strong. To demonstrate this fact, we calculated the distance matrix for the path metric of the tree and compared it to the distances calculated by ding-cf. On average, the tree path metric deviates only by $0.5\%$ per entry from the distances calculated by ding-cf with the largest relative difference being $2\%$ for the distance of *D. melanogaster* and *D. simulans*. We were able to further confirm this strong correspondence between the tree and the distance data via a split decomposition with SplitsTree4 in Additional file 1: Appendix D.1 [14, 15]. Overall, judging from these experiments, ding-cf looks promising as a distance measure for phylogenetic analyses.

However, we want to draw the reader’s attention to one possible pitfall of our method as a phylogenetic tool, namely that the fragmentation of the genome itself appears as a signal in the distance data. To emphasize this, let us pose a hypothetical extreme example: Consider a comparison between two assemblies ${\mathbb {A}},{\mathbb {B}}$ with *n* markers each, with a matching between all markers of ${\mathbb {A}}$ and ${\mathbb {B}}$. Suppose ${\mathbb {A}}$ is fully assembled into one chromosome and ${\mathbb {B}}$ fragments into *n* contigs of one marker. No matter the actual structure of the underlying (true) genome of ${\mathbb {B}}$, the DCJ distance between the assemblies ${\mathbb {A}}$ and ${\mathbb {B}}$ is always $n-1$. The size of this effect for practical levels of fragmentation needs to be investigated, particularly whether these problems could be exacerbated by biases in the assembly method used to arrive at the studied pair of genomes, such as might be the case for comparative assembly strategies.

**Fig. 15:**
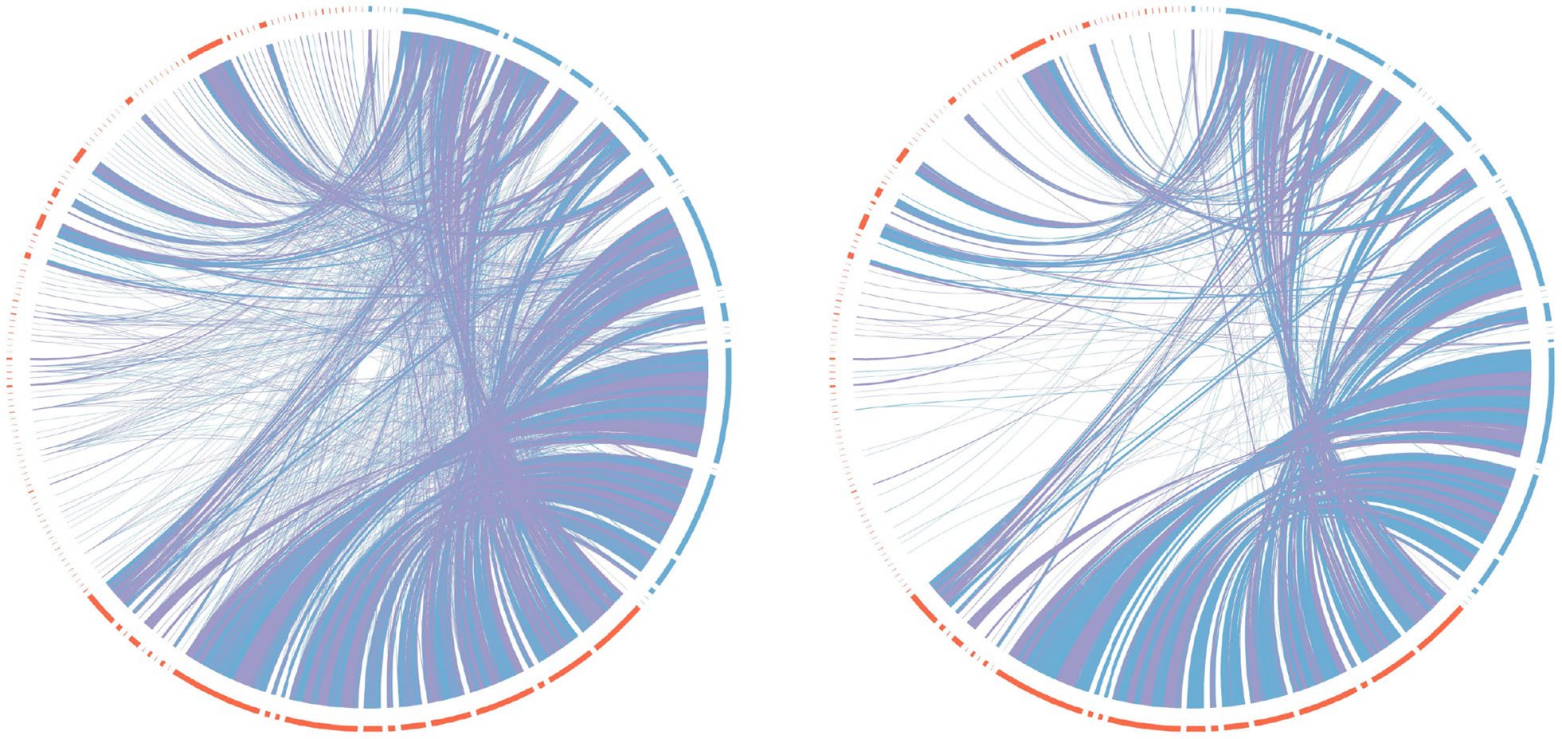
Circos plots for Contigs of *D. virilis* (red segments) and *D. mojavensis* (blue segments). Blue arcs show common markers with the same direction, purple arcs show common markers with different directions. On the left: before matching. On the right: after matching with ding-cf

*Refining Orthology to Match Contigs and Chromosomes.* We extracted the matchings from the ILP solutions calculated by gurobi and plotted them with Circos [16]. We show the matching between *D. virilis* and *D. mojavensis* in Fig. 15 as compared to just the marker matches identified by OrthoFinder. The plots for all other pairs can be found in Additional file 1: Appendix D.2. We see that even though there are some big rearrangements, such as inversions and transpositions as indicated by the arcs as well as an abundance of duplicates, the calculated matching identifies large stretches of matched markers in the same order and orientation as well as stretches of markers matched predominantly with markers from another such stretch. Depending on the particular definition of synteny, blocks such as these are known as *syntenic blocks* in the literature. While at this point we see no direct relation between the DCJ-indel model and models explicitly focusing on synteny, such as the syntenic distance in [17], we believe that refining orthologies using our ILP reveals important syntenic information.

For example, in many of the smaller contigs markers are matched predominantly to markers of one large contig of the other species. Matchings like this could therefore possibly be used to aid in improving very fragmented assemblies, given a sufficiently closely related and resolved reference genome.

## Conclusion

We presented a new, simpler distance formula for the DCJ-indel model. Using this distance formula, we were able to explain the previously unclear relationship between the BWS- and Compeau-conceptualizations of the DCJ-indel model. Furthermore, our formula is easily generalizeable to a performant ILP solution that enables the distance computation even for genomes fragmented into thousands of contigs. We have shown that a DCJ-indel analysis can be meaningful even with relatively fragmented genomes by applying the ILP to 11 *Drosophila* assemblies. From this we obtained a well resolved phylogeny with little noise in the distance data, indicating that our method could be well suited for distance based phylogenetic analyses provided the effect size of genome fragmentation in the particular use case can be bounded. We also showed that the ILP can be used to disambiguate orthologous and paralogous regions, which has potential use cases in orthology assignment and the finalization of fragmented assemblies.

Furthermore, we are confident that using this new formula, capping-free versions of other existing algorithms, such as for the family-free distance problem as in [8, 18] and parsimony problems as in [19] can be devised.

### Supplementary Information

**Additional file 1: **Supplementary Figures and Tables.

## Figures and Tables

**Algorithm 2:**
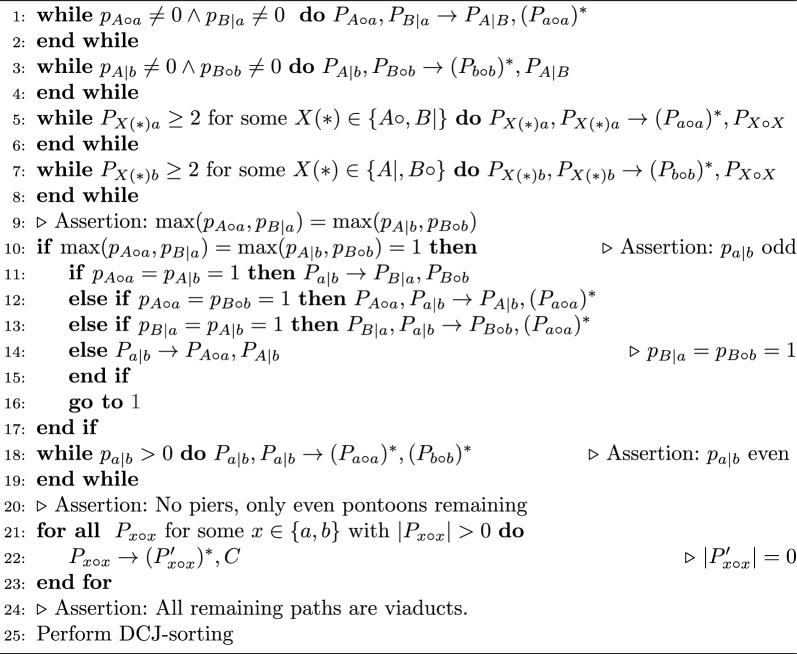
DCJ-indel sorting

## Data Availability

The ding-cf software and workflow are available at https://gitlab.ub.uni-bielefeld.de/gi/ding-cf. The preprocessed *Drosophila* data is available at https://uni-bielefeld.sciebo.de/s/06wGjSlvk7jeuWR. All *Drosophila* synteny plots are available at https://doi.org/10.6084/m9.figshare.24480892.v1.

## Appendix A Derivation of the distance formula

In this section, we derive the distance formula presented in the  "Relation of the BWS- and Compeau-Conceptualization" section. formally. We do this mostly by heavily referencing general statements about graphs like the MRD from [9], particularly from Section 3 there. We start by presenting an upper bound.

### Proposition 2

For two genomes ${\mathbb {A}}, {\mathbb {B}}$ with a resolved homology (${\mathop {\equiv }\limits ^{\star }}$) containing no circular singletons holds

$$\begin{aligned} \overline{d_{DCJ}^{id}} ({\mathbb {A}}, {\mathbb {B}},{\mathop {\equiv }\limits ^{\star }}) \ge n -c_{\circ } +\left\lceil {\frac{p_{a |b} + \max (p_{A \circ a},p_{B |a}) +\max (p_{A |b},p_{B \circ b}) - p_{A |B}}{2}}\right\rceil \end{aligned}$$

where $$n= |\{ (m,m') | m \in M_{{\mathbb {A}}}, m'\in M_{{\mathbb {B}}}, m{\mathop {\equiv }\limits ^{\star }} m' \}|$$.

We note that since the MRD is bipartite concerning extremity edges, parity and genomes of endpoints are determined by each other, that is ${\mathbb {P}}_{XX}={\mathbb {P}}_{X\circ X},{\mathbb {P}}_{Xx}={\mathbb {P}}_{X\circ x},{\mathbb {P}}_{xx}={\mathbb {P}}_{x\circ x}$ and ${\mathbb {P}}_{XY}={\mathbb {P}}_{X|Y},{\mathbb {P}}_{Xy}={\mathbb {P}}_{X|y},{\mathbb {P}}_{xy}={\mathbb {P}}_{x|y}$ for $\mathbb {X},\mathbb {Y}\in \{{\mathbb {A}},{\mathbb {B}}\}$ with $\mathbb {X}\ne \mathbb {Y}$. As a reminder, we use the following shorthands

$$\begin{aligned} F&:= n - c_{\circ } + \tilde{P}:= n- c_{\circ } +\left\lceil {\frac{\tilde{p}}{2}}\right\rceil \\&:= n -c_{\circ } + \left\lceil {\frac{p_{a |b} + \max (p_{A \circ a},p_{B |a}) + \max (p_{A |b},p_{B \circ b}) - p_{A |B}}{2}}\right\rceil \end{aligned}$$

for select formula terms. We start proving Proposition 2 by noticing that

### Proposition 3

For two genomes ${\mathbb {A}},{\mathbb {B}}$ with a resolved homology ($\equiv$) containing no circular singletons holds

$$\begin{aligned} {\mathbb {A}}\equiv \,{\mathbb {B}}\iff n -c_{\circ } + \left\lceil {\frac{p_{a |b} +\max (p_{A \circ a},p_{B |a}) + \max (p_{A |b}, p_{B \circ b}) - p_{A |B}}{2}}\right\rceil = 0. \end{aligned}$$

### Proof

If there are no singular markers, $F$ reduces to

$$\begin{aligned} n - c_{\circ } + \left\lceil { - \frac{p_{A |B}}{2}}\right\rceil = n - (c+ \left\lfloor {\frac{p_{A |B}}{2}}\right\rfloor ) {\mathop {=}\limits ^{p_{A |B} \text { even }}} n - (c + \frac{p_{A |B}}{2}), \end{aligned}$$

which is the DCJ distance formula by Bergeron et al. and therefore has already been shown to be 0 if and only if both genomes are equal [10]. If there are singular markers, obviously the two genomes cannot be equal, so the forward direction is trivial. For the backward direction, notice that every extremity of matched markers needs to either contribute to a 2-cycle or 1-path to reduce $n - c_\circ + \left\lceil {\frac{-p_{A|B}}{2}}\right\rceil$ to zero. Thus, every chromosome with at least one matched marker has a homologue. The only difference between the two genomes can be singleton chromosomes. Since our premise is that the genomes contain no circular singletons, only linear singletons remain. These, however, contain even piers $P_{A\circ a}$ or $P_{B\circ b}$ at their ends, thus $\max (p_{A\circ a},p_{A|b}) + \max (p_{B|a},p_{B\circ b}) > 0$ and with that $F> 0$, a contradiction. Therefore, both genomes must be equal. $\square$

We continue our proof of Proposition 2 by showing the influence of DCJ operations on the formula. To that end, we use the $\Delta _o$ operator used in [9], which denotes the difference in a quantity before and after operation *o*.

### Proposition 4

For any DCJ operation *d* holds

$$\begin{aligned} \Delta _d (n -c_{\circ } + \left\lceil {\frac{p_{a |b} + \max (p_{A \circ a},p_{B |a}) + \max (p_{A |b},p_{B \circ b}) - p_{A |B}}{2}}\right\rceil ) \ge -1. \end{aligned}$$

### Proof

Clearly, DCJ operations do not influence *n*. From [9] we know that $\Delta c_{\circ } \ge -1$ (see Corollary 2) and that none of the terms in the numerator can change if the number of cycles changes (see Observation 3 in [9]). We therefore only need to concern ourselves with $\tilde{P}$. We will show that its numerator $\tilde{p}$ can be reduced by at most 2. To that end, we first observe the way the maximizations in the formula behave. Clearly, the maximum will change at most as much as one of the its elements.$\square$

### Observation 1

For any operation *o* holds

$$\begin{aligned} \Delta _o \max (x,y)&\ge \Delta _o x \text { or}\\ \Delta _o \max (x,y)&\ge \Delta _o y \end{aligned}$$

Together with Corollary 1 from [9] we are able to derive the first bound for the numerator.

### Corollary 1

For a given DCJ operation *d*, there are $x\in \{p_{A\circ a},p_{B|a}\}$ and $y\in \{ p_{B|a},p_{B\circ b}\}$, for which hold

$$\begin{aligned} \Delta _d (p_{a |b} + \max (p_{A \circ a},p_{B |a}) +\max (p_{A |b},p_{B \circ b})) \ge \Delta _d (p_{a |b} +x + y) {\mathop {\ge }\limits ^{\text {Cor.~1}}} - 2. \end{aligned}$$

We see that the only way that $\tilde{p}$ could be reduced by more than two is if $\Delta p_{A|B} > 0$ and some other term is decreased at the same time. From Observation 5 from [9] we know that this cannot be $p_{a|b}$. From Observation 4 from [9] we know that $\Delta p_{A|B} \le 1$ if we decrease the other terms. The only remaining operations are then of the form $P_{Ay},P_{Bz} \rightarrow P_{AB},P_{yz}$. Clearly, any operation where $y=z$ will have $\Delta \tilde{p}= -2$. If $y\ne z$, the number of odd pontoons is increased, as seen also in Observation 6 of [9]. Thus, $\Delta \tilde{p}\ge -2$ and with that $\Delta \tilde{P}\ge -1$. This concludes our proof of Proposition 4. $\square$

We are left to examine the effect of indel operations. To that end, we need the definition of a *uni-indel* as defined in [9]. In short, uni-indels insert a single adjacency or delete a single marker. Regarding Observation 7 of [9], we see that a uni-indel either concatenates two piers or pontoons, thereby possibly creating a viaduct or creates a cycle from an even pontoon. From this follows that a uni-indel $i_1$ has either $\Delta _{i_1} c_{\circ } \le 1$ and $\Delta _{i_1} \tilde{P}= 0$ or $\Delta _{i_1}c_{\circ } = 0$ and $\Delta _{i_1} \tilde{P}\ge - 1$. Since none of the relevant component subsets contains (even) pontoons of length 0, we conclude using Observation 8 of [9]:

### Observation 2

For any deletion $\delta$ holds

$$\begin{aligned} \Delta _\delta F\ge -1 \end{aligned}$$

We will treat insertions in the same way as in Observation 9 of [9]. An insertion of a circular chromosome of length *l* has $\Delta n = l$, but each of the *l* uni-indels reduces $F$ by at most 1. Thus, we have $\Delta F\ge 0$ after applying the uni-indels, but before applying the DCJ operation. Since DCJ operations reduce $F$ by at most 1, we have

### Observation 3

For any insertion $\iota$ holds

$$\begin{aligned} \Delta _\iota F\ge -1. \end{aligned}$$

Together Observations 2 and 3 as well as Propositions 3 and 4, conclude our proof for the lower bound in Proposition 2.

To show that Theorem 1 holds, we need to show that this lower bound can be attained. To that end, we give a sorting procedure, in which each step decreases $F$ by 1. For the sake of simplicity, we will sort both genomes to an intermediate genome that contains no markers singular to ${\mathbb {A}}$ or ${\mathbb {B}}$. The advantage of this is that we do not need to consider in which genome we apply a DCJ operation and simply focus on the components involved. We can also use deletions only, which in this context are easier to conceptualize. Since each operation has an inverse, the sorting scenario from both genomes to an intermediate also describes a sorting scenario from ${\mathbb {A}}$ to ${\mathbb {B}}$. We give the sorting procedure in Algorithm 2.

Every step in the algorithm is conceived as a DCJ operation $X,Y\rightarrow W,Z$ transforming *X*, *Y* into *Y*, *Z*. This is not always possible without creating a circular singleton, which can only arise if an even pontoon of length 0 is created. In these cases, we have written the operation as $X,Y\rightarrow W,(Z)^*$ instead. If creating *Z* would generate a circular singleton, we simply replace the operation by the deletion $X,Y\rightarrow W$.

We now regard the assertions written as comments. Both assertions in Line 9 and 10 follow from a simple observation: Due to the fact that there is an even number of extremities of singular markers in each genome, there must be an even number of paths with an odd number of those extremities. Thus follows:

### Observation 4

$$\begin{aligned} p_{A\circ a}+p_{B|a}\equiv p_{a|b}\equiv p_{A|b}+p_{B\circ b} \mod 2 \end{aligned}$$

Due to the while-conditions in Lines 1 and 3, to reach these lines, one of the summands must be 0. Thus, we know that $p_{A\circ a}+p_{B|a} = \max (p_{A\circ a},p_{B|a})$ and due to the while-conditions in Lines 5 and 7, we know that $p_{A\circ a},p_{A|b},p_{B|a},p_{B\circ b}\in \{0,1\}$.

Due to the fact that for odd $p_{a\circ b}$ the algorithm starts from the beginning (see Line 16), it is clear that the assertion in Line 18 holds, although we should note that this goto-instruction is used at most once due to the fact that the parity of $p_{a|b}$ is only once changed in the if-branch. The remaining assertions are trivial and follow directly from previous while-conditions being false in order to reach the respective line.

**Table 3:** Changes to terms of $F$ by operations of Algorithm 2

Step	$-\Delta c_\circ$	$\Delta p_{a|b}$	$\Delta \max (p_{A\circ a},p_{B|a})$	$\Delta \max (p_{A|b},p_{B\circ b})$	$-\Delta p_{A|B}$
1	0	0	− 1	0	− 1
3	0	0	0	− 1	− 1
5	0	0	− 2	0	0
7	0	0	0	− 2	0
11	0	− 1	0	0	0
12	0	− 1	− 1	0	0
13	0	− 1	− 1	0	0
14	0	− 1	0	0	0
18	0	− 2	0	0	0
22	− 1	0	0	0	0

We see that almost every step decreases either $-c_\circ$ by 1 or the numerator $\tilde{p}$ by 2 while leaving other terms untouched. Therefore, these steps trivially reduce $F$ by 1. As a reference, we list the corresponding differences in Table 3. However, there are two problematic steps, namely 11 and 14, that reduce $p_{ab}$ by only 1 and do not reduce any other terms. To see how they nonetheless reduce $F$ by 1, we need an additional observation: Since each linear chromosome has two telomeres, the number of paths containing an odd number of telomeres in each genome, must me even, that is:

### Observation 5

$$\begin{aligned} p_{A\circ a}+p_{A|b} \equiv p_{A|B}\equiv p_{B|a}+p_{B\circ b}\mod 2 \end{aligned}$$

We thus know that for Lines 11, 14 the numerator $\tilde{p}$ before the operation is an odd number because of

$$\begin{aligned} p_{a|b} + p_{A\circ a} + p_{A|b} - p_{A|B} {\mathop {\equiv }\limits ^{Obs.~5}} p_{a|b} \equiv 1\mod 2 \end{aligned}$$

and thus changes by $-1$ to an even number, decreasing $\tilde{P}$ and therefore $F$ by 1.

## Footnotes

1 https://gitlab.ub.uni-bielefeld.de/gi/ding-cf.

2 https://gitlab.ub.uni-bielefeld.de/gi/ffs-dcj.
