# Supplementary material for: Recombinations, chains and caps: resolving problems with the DCJ-indel model
Source: Algorithms Mol Biol. 2024 Feb 27;19:8. doi: 10.1186/s13015-024-00253-7 (PMC10900646; doi:10.1186/s13015-024-00253-7)
Supplement: Supplementary file 1 — Additional file 1: Supplementary Figures and Tables. [file 13015_2024_253_MOESM1_ESM.pdf]

Appendix B Supplementary Figures and Tables for Subsection 3.1

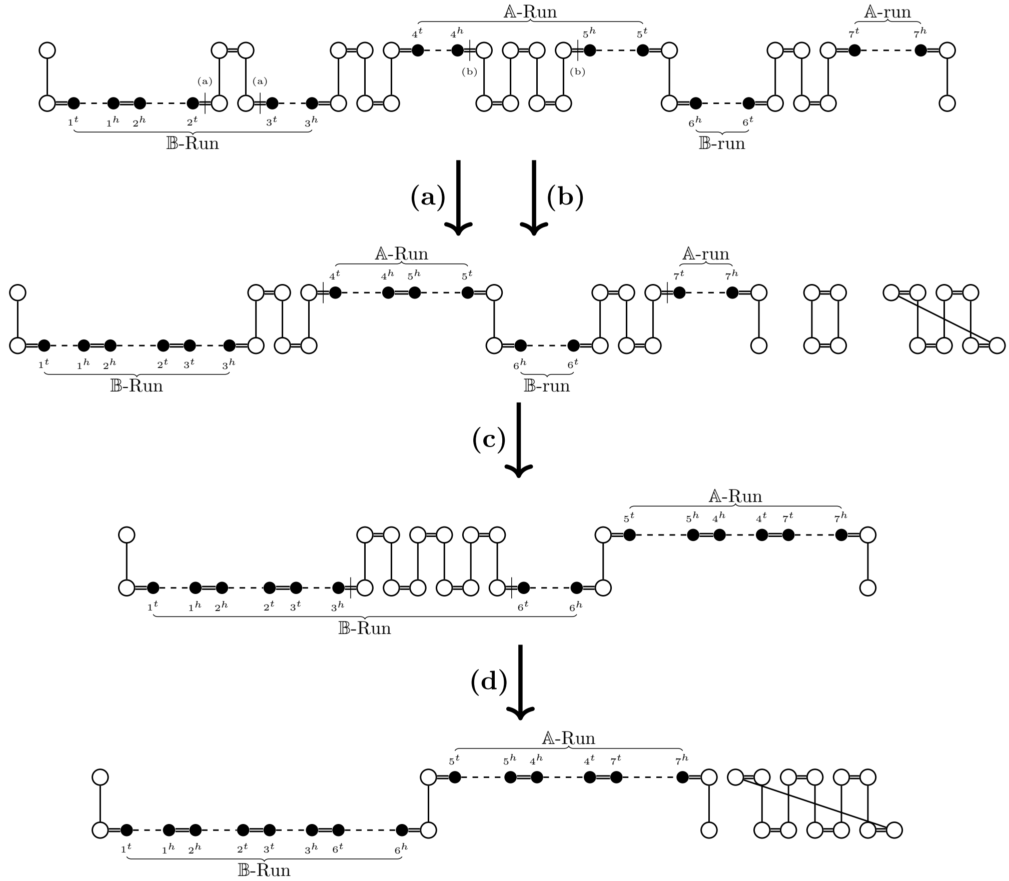

Fig. B1: Safe internal operations ((a) to (d)) in a bridge of type $P_{A|b}, P_{a|b}, P_{B|a}$ "accumulate" elements of a run ((a),(b) and (d)) or "cut in between runs" reducing their number by 2 as described in [11]. In the end, only the components $P_{A|b}, P_{a|b}$ and $P_{B|a}$ are not yet handled. Note also that the same sorting can be achieved after a completion as in Figure B2.

Table B2: The indel potential from [11] corresponds to the term $\tilde{P}$ in our distance formula if considered for a single bridge. Bridges with an odd number of extremities per genomes (i.e. odd bridges) were padded with an odd viaduct of length 1.

Path in [11]	$\Lambda(c)$	$\lambda(c) - \frac{p_c}{2}$ in [11]	Bridge X in reduced notation	$p_{a\|b}(X)$	$\max(p_{A\circ a}, p_{B\|a})(X)$	$\max(p_{A\|b}, p_{B\circ b})(X)$	$-p_{A\|B}(X)$	$\tilde{P}(X)$
$AA_{\mathcal{A}}$	k odd	$\left\lceil\frac{k+1}{2}\right\rceil - 0 = \frac{k+1}{2}$	$P_{A\circ a}, P_{A\circ a}$	$k-1$	2	0	0	$\left\lceil\frac{k+1}{2}\right\rceil = \frac{k+1}{2}$
$AA_{\mathcal{AB}}$	k even	$\left\lceil\frac{k+1}{2}\right\rceil - 0 = \frac{k+2}{2}$	$P_{A\circ a}, P_{a\|b}, P_{A\|b}$	$k-1$	1	1	0	$\left\lceil\frac{k+1}{2}\right\rceil = \frac{k+2}{2}$
$AA_{\mathcal{B}}$	k odd	$\left\lceil\frac{k+1}{2}\right\rceil - 0 = \frac{k+1}{2}$	$P_{A\|b}, P_{A\|b}$	$k-1$	1	1	0	$\left\lceil\frac{k+1}{2}\right\rceil = \frac{k+1}{2}$
$AB_{\mathcal{A}}$	k odd	$\left\lceil\frac{k+1}{2}\right\rceil - 1 = \frac{k-1}{2}$	$(P_{A\circ a}), (P_{B\|a})$	$k-1$	1	0	-1	$\left\lceil\frac{k-1}{2}\right\rceil = \frac{k-1}{2}$
$AB_{\mathcal{AB}}$	k even	$\left\lceil\frac{k+1}{2}\right\rceil - 1 = \frac{k}{2}$	$P_{A\circ a}, P_{a\|b}, P_{B\circ b}$	$k-1$	1	1	-1	$\left\lceil\frac{k}{2}\right\rceil = \frac{k}{2}$
$AB_{\mathcal{BA}}$	k even	$\left\lceil\frac{k+1}{2}\right\rceil - 1 = \frac{k}{2}$	$P_{A\|b}, P_{a\|b}, P_{B\|a}$	$k-1$	1	1	-1	$\left\lceil\frac{k}{2}\right\rceil = \frac{k}{2}$
$AB_{\mathcal{B}}$	k odd	$\left\lceil\frac{k+1}{2}\right\rceil - 1 = \frac{k-1}{2}$	$(P_{A\|b}), (P_{B\circ b})$	$k-1$	0	1	-1	$\left\lceil\frac{k-1}{2}\right\rceil = \frac{k-1}{2}$
$BB_{\mathcal{A}}$	k odd	$\left\lceil\frac{k+1}{2}\right\rceil - 0 = \frac{k+1}{2}$	$P_{B\|a}, P_{B\|a}$	$k-1$	2	0	0	$\left\lceil\frac{k+1}{2}\right\rceil = \frac{k+1}{2}$
$BB_{\mathcal{AB}}$	k even	$\left\lceil\frac{k+1}{2}\right\rceil - 0 = \frac{k+2}{2}$	$P_{B\|a}, P_{a\|b}, P_{B\circ b}$	$k-1$	1	1	0	$\left\lceil\frac{k+1}{2}\right\rceil = \frac{k+2}{2}$
$BB_{\mathcal{B}}$	k odd	$\left\lceil\frac{k+1}{2}\right\rceil - 0 = \frac{k+1}{2}$	$P_{B\circ b}, P_{B\circ b}$	$k-1$	0	2	0	$\left\lceil\frac{k+1}{2}\right\rceil = \frac{k+1}{2}$

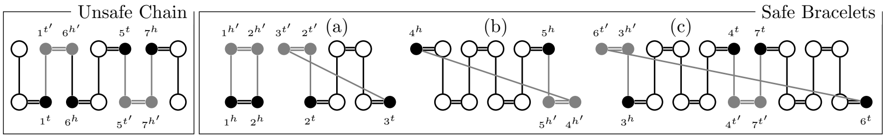

Fig. B2: Forming bracelets and chains (as in [5]) from the components of the bridge of type $P_{A|b}, P_{a|b}, P_{B|a}$ of Figure B1 leaves only $P_{A|b}, P_{a|b}$ and $P_{B|a}$ in an unsafe chain. Note how bracelets (a),(b) and (c) create exactly the same adjacencies as part of indel groups as operations (a),(b) and (c) in Figure B1 respectively.

Table B3: Path Recombinations used in Recombination Groups as listed in Tables 1 and 2 of [11] are explained by safe operations between unsaturated components. The concrete effect on the distance as opposed to a separate sorting is explained by the differences of $\tilde{P}$ in two graphs containing the respective sources separately as opposed to one graph containing both. Paddings are administered as in Table B2.

	Source 1	Bridge X_1	$\tilde{P}(X_1)$	Source 2	Bridge X_2	$\tilde{P}_2(X_2)$	Safe operation(s)	Uns. Comp.	$\tilde{P}(X_1 \cup X_2)$	$\Delta\tilde{P}$
	$AA_{\mathcal{AB}}$	$P_{Aoa}, P_{a\|b}, P_{A\|b}$	$\frac{k_1+2}{2}$	$BB_{\mathcal{AB}}$	$P_{B\|a}, P_{a\|b}, P_{Bob}$	$\frac{k_2+2}{2}$	$P_{Aoa}, P_{B\|a} \rightarrow P_{A\|B}, P_{aoa}$, $P_{a\|b}, P_{a\|b} \rightarrow P_{aoa}, P_{bob}$, $P_{A\|b}, P_{Bob} \rightarrow P_{A\|B}, P_{bob}$	-	$\frac{k_1+k_2}{2}$	−2
	$AA_{\mathcal{A}}$	P_{Aoa}, P_{Aoa}	$\frac{k_1+1}{2}$	$BB_{\mathcal{AB}}$	$P_{B\|a}, P_{a\|b}, P_{Bob}$	$\frac{k_2+2}{2}$	$P_{Aoa}, P_{B\|a} \rightarrow P_{A\|B}, P_{aoa}$	$P_{Aoa}, P_{a\|b}, P_{Bob}$	$\frac{k_1+k_2+1}{2}$	−1
	$BB_{\mathcal{A}}$	$P_{B\|a}, P_{B\|a}$	$\frac{k_1+1}{2}$	$AA_{\mathcal{AB}}$	$P_{Aoa}, P_{a\|b}, P_{A\|b}$	$\frac{k_2+2}{2}$	$P_{Aoa}, P_{B\|a} \rightarrow P_{A\|B}, P_{aoa}$	$P_{B\|a}, P_{a\|b}, P_{A\|b}$	$\frac{k_1+k_2+1}{2}$	−1
	$AA_{\mathcal{B}}$	$P_{A\|b}, P_{A\|b}$	$\frac{k_1+1}{2}$	$BB_{\mathcal{AB}}$	$P_{B\|a}, P_{a\|b}, P_{Bob}$	$\frac{k_2+2}{2}$	$P_{A\|b}, P_{Bob} \rightarrow P_{A\|B}, P_{bob}$	$P_{B\|a}, P_{a\|b}, P_{A\|b}$	$\frac{k_1+k_2+1}{2}$	−1
	$BB_{\mathcal{B}}$	P_{Bob}, P_{Bob}	$\frac{k_1+1}{2}$	$AA_{\mathcal{AB}}$	$P_{Aoa}, P_{a\|b}, P_{A\|b}$	$\frac{k_2+2}{2}$	$P_{A\|b}, P_{Bob} \rightarrow P_{A\|B}, P_{bob}$	$P_{Aoa}, P_{a\|b}, P_{Bob}$	$\frac{k_1+k_2+1}{2}$	−1
	$AA_{\mathcal{A}}$	P_{Aoa}, P_{Aoa}	$\frac{k_1+1}{2}$	$BB_{\mathcal{A}}$	$P_{B\|a}, P_{B\|a}$	$\frac{k_2+1}{2}$	$P_{Aoa}, P_{B\|a} \rightarrow P_{A\|B}, P_{aoa}$	-	$\frac{k_1+k_2}{2}$	−1
	$AA_{\mathcal{B}}$	$P_{A\|b}, P_{A\|b}$	$\frac{k_1+1}{2}$	$BB_{\mathcal{B}}$	P_{Bob}, P_{Bob}	$\frac{k_2+1}{2}$	$P_{A\|b}, P_{Bob} \rightarrow P_{A\|B}, P_{bob}$	-	$\frac{k_1+k_2}{2}$	−1
	$AA_{\mathcal{AB}}$	$P_{Aoa}, P_{a\|b}, P_{A\|b}$	$\frac{k_1+2}{2}$	$AA_{\mathcal{AB}}$	$P_{Aoa}, P_{a\|b}, P_{A\|b}$	$\frac{k_2+2}{2}$	$P_{a\|b}, P_{a\|b} \rightarrow P_{aoa}, P_{bob}$	$P_{Aoa}, P_{Aoa}, P_{A\|b}, P_{A\|b}$	$\frac{k_1+k_2+2}{2}$	−1
	$BB_{\mathcal{AB}}$	$P_{B\|a}, P_{a\|b}, P_{Bob}$	$\frac{k_1+2}{2}$	$BB_{\mathcal{AB}}$	$P_{B\|a}, P_{a\|b}, P_{Bob}$	$\frac{k_2+2}{2}$	$P_{a\|b}, P_{a\|b} \rightarrow P_{aoa}, P_{bob}$	$P_{B\|a}, P_{B\|a}, P_{Bob}, P_{Bob}$	$\frac{k_1+k_2+2}{2}$	−1
	$AA_{\mathcal{AB}}$	$P_{Aoa}, P_{a\|b}, P_{A\|b}$	$\frac{k_1+2}{2}$	$AB_{\mathcal{AB}}$	$P_{Aoa}, P_{a\|b}, P_{Bob}$	$\frac{k_2}{2}$	$P_{a\|b}, P_{a\|b} \rightarrow P_{aoa}, P_{bob}$, $P_{A\|b}, P_{Bob} \rightarrow P_{A\|B}, P_{bob}$	P_{Aoa}, P_{Aoa}	$\frac{k_1+k_2}{2}$	−1
	$AA_{\mathcal{AB}}$	$P_{Aoa}, P_{a\|b}, P_{A\|b}$	$\frac{k_1+2}{2}$	$AB_{\mathcal{BA}}$	$P_{A\|b}, P_{a\|b}, P_{B\|a}$	$\frac{k_2}{2}$	$P_{Aoa}, P_{B\|a} \rightarrow P_{A\|B}, P_{aoa}$, $P_{a\|b}, P_{a\|b} \rightarrow P_{aoa}, P_{bob}$	$P_{A\|b}, P_{A\|b}$	$\frac{k_1+k_2}{2}$	−1
	$BB_{\mathcal{AB}}$	$P_{B\|a}, P_{a\|b}, P_{Bob}$	$\frac{k_1+2}{2}$	$AB_{\mathcal{AB}}$	$P_{Aoa}, P_{a\|b}, P_{Bob}$	$\frac{k_2}{2}$	$P_{Aoa}, P_{B\|a} \rightarrow P_{A\|B}, P_{aoa}$, $P_{a\|b}, P_{a\|b} \rightarrow P_{aoa}, P_{bob}$	P_{Bob}, P_{Bob}	$\frac{k_1+k_2}{2}$	−1
	$BB_{\mathcal{AB}}$	$P_{B\|a}, P_{a\|b}, P_{Bob}$	$\frac{k_1+2}{2}$	$AB_{\mathcal{BA}}$	$P_{A\|b}, P_{a\|b}, P_{B\|a}$	$\frac{k_2}{2}$	$P_{a\|b}, P_{a\|b} \rightarrow P_{aoa}, P_{bob}$, $P_{A\|b}, P_{Bob} \rightarrow P_{A\|B}, P_{bob}$	$P_{B\|a}, P_{B\|a}$	$\frac{k_1+k_2}{2}$	−1
	$AB_{\mathcal{AB}}$	$P_{Aoa}, P_{a\|b}, P_{Bob}$	$\frac{k_1}{2}$	$AB_{\mathcal{BA}}$	$P_{A\|b}, P_{a\|b}, P_{B\|a}$	$\frac{k_2}{2}$	$P_{Aoa}, P_{B\|a} \rightarrow P_{A\|B}, P_{aoa}$, $P_{a\|b}, P_{a\|b} \rightarrow P_{aoa}, P_{bob}$, $P_{A\|b}, P_{Bob} \rightarrow P_{A\|B}, P_{bob}$	-	$\frac{k_1+k_2-2}{2}$	−1
	$AA_{\mathcal{A}}$	P_{Aoa}, P_{Aoa}	$\frac{k_1+1}{2}$	$AB_{\mathcal{BA}}$	$P_{A\|b}, P_{a\|b}, P_{B\|a}$	$\frac{k_2}{2}$	$P_{Aoa}, P_{B\|a} \rightarrow P_{A\|B}, P_{aoa}$	$P_{Aoa}, P_{a\|b}, P_{A\|b}$	$\frac{k_1+k_2+1}{2}$	0
	$AA_{\mathcal{B}}$	$P_{A\|b}, P_{A\|b}$	$\frac{k_1+1}{2}$	$AB_{\mathcal{AB}}$	$P_{Aoa}, P_{a\|b}, P_{Bob}$	$\frac{k_2}{2}$	$P_{A\|b}, P_{Bob} \rightarrow P_{A\|B}, P_{bob}$	$P_{Aoa}, P_{a\|b}, P_{A\|b}$	$\frac{k_1+k_2+1}{2}$	0
	$BB_{\mathcal{A}}$	$P_{B\|a}, P_{B\|a}$	$\frac{k_1+1}{2}$	$AB_{\mathcal{AB}}$	$P_{Aoa}, P_{a\|b}, P_{Bob}$	$\frac{k_2}{2}$	$P_{Aoa}, P_{B\|a} \rightarrow P_{A\|B}, P_{aoa}$	$P_{B\|a}, P_{a\|b}, P_{Bob}$	$\frac{k_1+k_2+1}{2}$	0
	$BB_{\mathcal{B}}$	P_{Bob}, P_{Bob}	$\frac{k_1+1}{2}$	$AB_{\mathcal{BA}}$	$P_{A\|b}, P_{a\|b}, P_{B\|a}$	$\frac{k_2}{2}$	$P_{A\|b}, P_{Bob} \rightarrow P_{A\|B}, P_{bob}$	$P_{B\|a}, P_{a\|b}, P_{Bob}$	$\frac{k_1+k_2+1}{2}$	0
⋆	$AA_{\mathcal{A}}$	P_{Aoa}, P_{Aoa}	$\frac{k_1+1}{2}$	$BB_{\mathcal{B}}$	P_{Bob}, P_{Bob}	$\frac{k_2+1}{2}$		$P_{Aoa}, P_{Aoa}, P_{Bob}, P_{Bob}$	$\frac{k_1+k_2+2}{2}$	0
⋆	$AA_{\mathcal{B}}$	$P_{A\|b}, P_{A\|b}$	$\frac{k_1+1}{2}$	$BB_{\mathcal{A}}$	$P_{B\|a}, P_{B\|a}$	$\frac{k_2+1}{2}$		$P_{B\|a}, P_{B\|a}, P_{A\|b}, P_{A\|b}$	$\frac{k_1+k_2+2}{2}$	0
	$AB_{\mathcal{AB}}$	$P_{Aoa}, P_{a\|b}, P_{Bob}$	$\frac{k_1}{2}$	$AB_{\mathcal{AB}}$	$P_{Aoa}, P_{a\|b}, P_{Bob}$	$\frac{k_2}{2}$	$P_{a\|b}, P_{a\|b} \rightarrow P_{aoa}, P_{bob}$	$P_{Aoa}, P_{Aoa}, P_{Bob}, P_{Bob}$	$\frac{k_1+k_2}{2}$	0
	$AB_{\mathcal{BA}}$	$P_{A\|b}, P_{a\|b}, P_{B\|a}$	$\frac{k_1}{2}$	$AB_{\mathcal{BA}}$	$P_{A\|b}, P_{a\|b}, P_{B\|a}$	$\frac{k_2}{2}$	$P_{a\|b}, P_{a\|b} \rightarrow P_{aoa}, P_{bob}$	$P_{B\|a}, P_{B\|a}, P_{A\|b}, P_{A\|b}$	$\frac{k_1+k_2}{2}$	0

Table B4: Recombination groups from [11] can be facilitated entirely by safe operations. In all but 4 cases, resultants either contain no further unsaturated components or if so, no further safe operations are available on the unsaturated components as a bridge containing the respective partner would have been used in an operation higher in the table (last column). Exceptions are the groups $S4, S5, S6, S7$ (marked with star). In these cases, the resultant $AB_{\mathcal{AB}}$ $(AB_{\mathcal{BA}})$ could still be safely recombined with $AB_{\mathcal{AB}}$ $(AB_{\mathcal{BA}})$, but this recombination is only neutral (see Table B3).

	Sources	Reusable Resultants	Unsat. after safe internal ops.					Unsat. after all safe ops. in group					No partner due to
			$A\circ a$	$B\vert a$	$a\vert b$	$A\vert b$	$B\circ b$	$A\circ a$	$B\vert a$	$p_{a\vert b}$	$A\vert b$	$B\circ b$	
P													
1	$AA_{\mathcal{AB}} + BB_{\mathcal{AB}}$		1	1	2	1	1	0	0	0	0	0	
Q													
1	$2AA_{\mathcal{AB}} + BB_{\mathcal{A}} + BB_{\mathcal{B}}$		2	2	2	2	2	0	0	0	0	0	
2	$2BB_{\mathcal{AB}} + AA_{\mathcal{A}} + AA_{\mathcal{B}}$		2	2	2	2	2	0	0	0	0	0	
T													
1	$AA_{\mathcal{AB}} + BB_{\mathcal{A}} + AB_{\mathcal{AB}}$		2	2	2	1	1	0	0	0	0	0	
2	$AA_{\mathcal{AB}} + BB_{\mathcal{B}} + AB_{\mathcal{BA}}$		1	1	2	2	2	0	0	0	0	0	
3	$BB_{\mathcal{AB}} + AA_{\mathcal{A}} + AB_{\mathcal{BA}}$		2	2	2	1	1	0	0	0	0	0	
4	$BB_{\mathcal{AB}} + AA_{\mathcal{B}} + AB_{\mathcal{AB}}$		1	1	2	2	2	0	0	0	0	0	
5	$2AA_{\mathcal{AB}} + BB_{\mathcal{A}}$	$AA_{\mathcal{B}}$	2	2	2	2	0	0	0	0	2	0	$P_{A\vert b} \to T1, Q1, P1;$
6	$2AA_{\mathcal{AB}} + BB_{\mathcal{B}}$	$AA_{\mathcal{A}}$	2	0	2	2	2	2	0	0	0	0	$P_{A\circ a} \to T2, Q1, P1;$
7	$2BB_{\mathcal{AB}} + AA_{\mathcal{A}}$	$BB_{\mathcal{B}}$	2	2	2	0	2	0	0	0	0	2	$P_{B\circ b} \to Q2, P1, T3;$
8	$2BB_{\mathcal{AB}} + AA_{\mathcal{B}}$	$BB_{\mathcal{A}}$	0	2	2	2	2	0	2	0	0	0	$P_{B\vert a} \to Q2, P1, T4;$
S													
1	$AA_{\mathcal{A}} + BB_{\mathcal{A}}$		2	2	0	0	0	0	0	0	0	0	
2	$AA_{\mathcal{B}} + BB_{\mathcal{B}}$		0	0	0	2	2	0	0	0	0	0	
3	$AB_{\mathcal{AB}} + AB_{\mathcal{BA}}$		1	1	2	1	1	0	0	0	0	0	
4	$AA_{\mathcal{AB}} + BB_{\mathcal{A}}$	$AB_{\mathcal{BA}}$	1	2	1	1	0	0	1	1	1	0	$P_{B\vert a} \to S1, T5, T1;$<br>$\star P_{a\vert b} \to T5, T1, P1;$<br>$\star P_{A\vert b} \to T1, P1;$
5	$AA_{\mathcal{AB}} + BB_{\mathcal{B}}$	$AB_{\mathcal{AB}}$	1	0	1	1	2	1	0	1	0	1	$P_{A\circ a} \to T2, S4, P1;$<br>$\star P_{a\vert b} \to T6, T2, P1;$<br>$P_{B\circ b} \to S2, T6, T2;$
6	$BB_{\mathcal{AB}} + AA_{\mathcal{A}}$	$AB_{\mathcal{AB}}$	2	1	1	0	1	1	0	1	0	1	$P_{A\circ a} \to T3, S1, T7;$<br>$\star P_{a\vert b} \to P1, T3, T7;$<br>$\star P_{B\circ b} \to P1, T3;$
7	$BB_{\mathcal{AB}} + AA_{\mathcal{B}}$	$AB_{\mathcal{BA}}$	0	1	1	2	1	0	1	1	1	0	$P_{B\vert a} \to S6, P1, T4;$<br>$\star P_{a\vert b} \to P1, T4, T8;$<br>$P_{A\vert b} \to T4, S2, T8;$
8	$AA_{\mathcal{AB}} + AB_{\mathcal{AB}}$	$AA_{\mathcal{A}}$	2	0	2	1	1	2	0	0	0	0	$P_{A\circ a} \to S3, T1, P1;$
9	$AA_{\mathcal{AB}} + AB_{\mathcal{BA}}$	$AA_{\mathcal{B}}$	1	1	2	2	0	0	0	0	2	0	$P_{A\vert b} \to S3, T2, P1;$
10	$BB_{\mathcal{AB}} + AB_{\mathcal{AB}}$	$BB_{\mathcal{B}}$	1	1	2	0	2	0	0	0	0	2	$P_{B\circ b} \to T4, P1, S3;$
11	$BB_{\mathcal{AB}} + AB_{\mathcal{BA}}$	$BB_{\mathcal{A}}$	0	2	2	1	1	0	2	0	0	0	$P_{B\vert a} \to T3, P1, S3;$
12	$2AA_{\mathcal{AB}}$	$AB_{\mathcal{A}} + AA_{\mathcal{B}}$	2	0	2	2	0	2	0	0	2	0	$P_{A\circ a} \to S9, T5, P1;$<br>$P_{A\vert b} \to S8, T6, P1;$
13	$2BB_{\mathcal{AB}}$	$BB_{\mathcal{A}} + BB_{\mathcal{B}}$	0	2	2	0	2	0	2	0	0	2	$P_{B\vert a} \to T7, P1, S10;$<br>$P_{B\circ b} \to T8, P1, S11;$
M													
1	$2AB_{\mathcal{AB}} + AA_{\mathcal{B}} + BB_{\mathcal{A}}$		2	2	2	2	2	0	0	0	0	0	
2	$2AB_{\mathcal{BA}} + AA_{\mathcal{A}} + BB_{\mathcal{B}}$		2	2	2	2	2	0	0	0	0	0	
N													
1	$AB_{\mathcal{AB}} + AA_{\mathcal{B}} + BB_{\mathcal{A}}$	$AB_{\mathcal{BA}}$	1	2	1	2	1	0	1	1	1	0	$P_{B\vert a} \to S1, T1, M1;$<br>$P_{a\vert b} \to T1, M1, S3, T4;$<br>$P_{A\vert b} \to M1, S2, T4;$
2	$AB_{\mathcal{BA}} + AA_{\mathcal{A}} + BB_{\mathcal{B}}$	$AB_{\mathcal{AB}}$	2	1	1	1	2	1	0	1	0	1	$P_{A\circ a} \to M2, S1, T3;$<br>$P_{a\vert b} \to T2, S3, M2, T3;$<br>$P_{B\circ b} \to S2, T2, M2;$
3	$2AB_{\mathcal{AB}} + AA_{\mathcal{B}}$	$AA_{\mathcal{A}}$	2	0	2	2	2	2	0	0	0	0	$P_{A\circ a} \to S3, M1, T4;$
4	$2AB_{\mathcal{AB}} + BB_{\mathcal{A}}$	$BB_{\mathcal{B}}$	2	2	2	0	2	0	0	0	0	2	$P_{B\circ b} \to M1, T1, S3;$
5	$2AB_{\mathcal{BA}} + AA_{\mathcal{A}}$	$AA_{\mathcal{B}}$	2	2	2	2	0	0	0	0	2	0	$P_{A\vert b} \to S3, M2, T3;$
6	$2AB_{\mathcal{BA}} + BB_{\mathcal{B}}$	$BB_{\mathcal{A}}$	0	2	2	2	2	0	2	0	0	0	$P_{B\vert a} \to M2, T2, S3;$

Appendix C Additional Performance Test Data

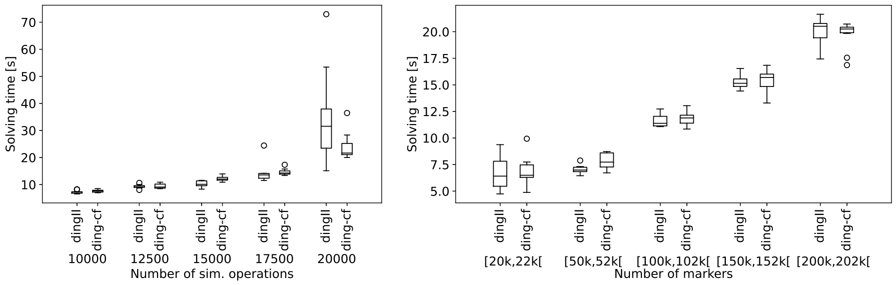

Fig. C3: `dingII` and `ding-cf` evaluated on simulated genomes with the same parameters as described in Section 5 with a single linear chromosome as the root genome. On the left, the number of simulated operations is increased from 10,000 to 20,000 in steps of 2500. On the right, the number of markers is increased from 10,000 and 2500 to 100,000 in steps of 25,000.

The further performance test data of Figure C3 indicates that `ding-cf` behaves similarly as `ding` regarding the number of operations and markers (compare to [7]), even outperforming `ding` on harder problem instances. The outliers for the smallest number of markers in Figure C3 indicates that the number of operations alone might not be such a significant factor, but rather the number of operations relative to the size of the genome.

Appendix D Supplementary Drosophila data

Table D5: Statistics about drosophila genomes after processing to unimog format.

Taxon	Assembly	#markers	#lin. chrom.	# circ. chrom.
D. ananassae	GCF_017639315.1	13838	100	0
D. erecta	GCF_003286155.1	12898	52	0
D. grimshawi	GCF_018153295.1	12108	41	0
D. melanogaster	GCF_000001215.4	13083	11	1
D. mojavensis	GCF_018153725.1	12464	35	0
D. persimilis	GCF_003286085.1	13630	211	0
D. sechellia	GCF_004382195.1	13189	76	0
D. simulans	GCF_016746395.2	13336	32	0
D. virilis	GCF_003285735.1	12821	131	0
D. willistoni	GCF_018902025.1	13901	357	0
D. yakuba	GCF_016746365.2	13306	26	1

Table D6: Statistics of all pairwise `ding-cf` runs with *lr* the number of linear, *cr* the number of circular chromosomes, mxfam the size of the largest family, #ambf the number of ambiguous families, # amb average number of markers of ambiguous families and *N* the total number of markers.

Pair	*lr*	*cr*	mxfam	#ambf	#amb	*N*	dist	gap	time
D. ananassae-D. erecta	152	0	22	783	1660	26736	2914	0.4461%	43200.24
D. ananassae-D. grimshawi	141	0	20	831	1679	25946	4363	0.2292%	43200.24
D. ananassae-D. melanogaster	111	1	25	752	1679	26921	2875	0.8696%	43200.06
D. ananassae-D. mojavensis	135	0	50	832	1736.5	26302	4369	0.2289%	43200.25
D. ananassae-D. persimilis	311	0	29	1073	2284.5	27468	3903	0.5380%	43200.31
D. ananassae-D. sechellia	176	0	49	808	1756.5	27027	3001	0.1333%	43200.28
D. ananassae-D. simulans	132	0	20	789	1783	27174	2999	0.9003%	43200.26
D. ananassae-D. virilis	231	0	20	1023	2051.5	26659	4407	0.4538%	43200.27
D. ananassae-D. willistoni	457	0	81	997	2306.5	27739	5153	0.4463%	43200.31
D. ananassae-D. yakuba	126	1	20	777	1769.5	27144	3001	0.7997%	43200.26
D. erecta-D. grimshawi	93	0	22	740	1454.5	25006	4243	0.0471%	43200.25
D. erecta-D. melanogaster	63	1	25	621	1420	25981	900	0.5556%	43200.32
D. erecta-D. mojavensis	87	0	22	714	1409.5	25362	4253	0.0941%	43200.24
D. erecta-D. persimilis	263	0	29	983	2038	26528	3715	0.1615%	43200.32
D. erecta-D. sechellia	128	0	30	667	1471	26087	1033	0.0000%	126.79
D. erecta-D. simulans	84	0	22	642	1456	26234	1025	0.0976%	43200.25
D. erecta-D. virilis	183	0	22	925	1788.5	25719	4264	0.2111%	43200.27
D. erecta-D. willistoni	409	0	22	869	1796	26799	5004	0.2598%	43200.27
D. erecta-D. yakuba	78	1	22	619	1424	26204	959	0.2086%	43200.27
D. grimshawi-D. melanogaster	52	1	25	695	1428.5	25191	4211	0.3087%	43200.24
D. grimshawi-D. mojavensis	76	0	16	722	1526.5	24572	2221	0.4953%	43200.24
D. grimshawi-D. persimilis	252	0	29	984	2014	25738	4376	0.2514%	43200.25
D. grimshawi-D. sechellia	117	0	30	745	1483	25297	4313	0.0000%	367.95
D. grimshawi-D. simulans	73	0	18	715	1467	25444	4294	0.0932%	43200.23
D. grimshawi-D. virilis	172	0	20	933	1902	24929	2240	0.4911%	43200.26
D. grimshawi-D. willistoni	398	0	18	885	1818.5	26009	5052	0.1584%	43200.28
D. grimshawi-D. yakuba	67	1	20	707	1455	25414	4267	0.1640%	43200.24
D. melanogaster-D. mojavensis	46	1	25	682	1409	25547	4218	0.1660%	43200.23
D. melanogaster-D. persimilis	222	1	29	945	2035	26713	3704	0.4320%	43200.29
D. melanogaster-D. sechellia	87	1	30	604	1434.5	26272	779	0.0000%	39.53
D. melanogaster-D. simulans	43	1	25	581	1453	26419	719	0.0000%	135.49
D. melanogaster-D. virilis	142	1	25	886	1769.5	25904	4278	0.1403%	43200.29
D. melanogaster-D. willistoni	368	1	25	831	1804.5	26984	5003	0.4197%	43200.27
D. melanogaster-D. yakuba	37	2	25	589	1437.5	26389	902	0.0000%	1081.82
D. mojavensis-D. persimilis	246	0	29	982	2017	26094	4399	0.1137%	43200.30
D. mojavensis-D. sechellia	111	0	30	729	1477.5	25653	4323	0.0000%	714.61
D. mojavensis-D. simulans	67	0	19	710	1482.5	25800	4321	0.1389%	43200.27
D. mojavensis-D. virilis	166	0	20	886	1862.5	25285	1866	0.4823%	43200.25
D. mojavensis-D. willistoni	392	0	29	883	1884.5	26365	5102	0.2940%	43200.28
D. mojavensis-D. yakuba	61	1	24	688	1458.5	25770	4300	0.0465%	43200.26
D. persimilis-D. sechellia	287	0	30	990	2071.5	26819	3806	0.1314%	43200.28
D. persimilis-D. simulans	243	0	29	958	2053.5	26966	3806	0.3153%	43200.28
D. persimilis-D. virilis	342	0	29	1175	2375	26451	4457	0.4936%	43200.30
D. persimilis-D. willistoni	568	0	29	1144	2423.5	27531	5197	0.3848%	43200.31
D. persimilis-D. yakuba	237	1	29	945	2033	26936	3774	0.3180%	43200.30
D. sechellia-D. simulans	108	0	30	628	1547.5	26525	768	0.0000%	4328.75
D. sechellia-D. virilis	207	0	30	934	1812	26010	4345	0.0000%	19154.21
D. sechellia-D. willistoni	433	0	30	883	1878	27090	5097	0.1766%	43200.30
D. sechellia-D. yakuba	102	1	30	634	1492.5	26495	1075	0.0000%	466.19
D. simulans-D. virilis	163	0	20	912	1826.5	26157	4366	0.3436%	43200.25
D. simulans-D. willistoni	389	0	29	859	1891.5	27237	5114	0.3911%	43200.28
D. simulans-D. yakuba	58	1	24	630	1595.5	26642	1029	1.4577%	43200.28
D. virilis-D. willistoni	488	0	56	1048	2185	26722	5123	0.3514%	43200.29
D. virilis-D. yakuba	157	1	20	890	1796	26127	4336	0.2537%	43200.29
D. willistoni-D. yakuba	383	1	32	849	1910	27207	5085	0.5310%	43200.25

D.1 Evaluating the Distance Matrix

To estimate the strength of the phylogenetic signal in the obtained distance data of Table D6, we compared it to the path metric of the reconstructed tree, which we give here in Newick format for reference:

```
(((ananassae:1537.6125,(((grimshawi:1095.78125,(mojavensis:915.77777778,
virilis:950.22222222):201.71875):1035.83928571,willistoni:2931.16071429):
309.89583333,persimilis:1948.47916667):396.2625):921.08007813,
erecta:454.70117188):18.54882813,(melanogaster:309.2890625,
(sechellia:398.18359375,simulans:369.81640625):55.7109375):
104.75195313,yakuba:497.74804688);
```

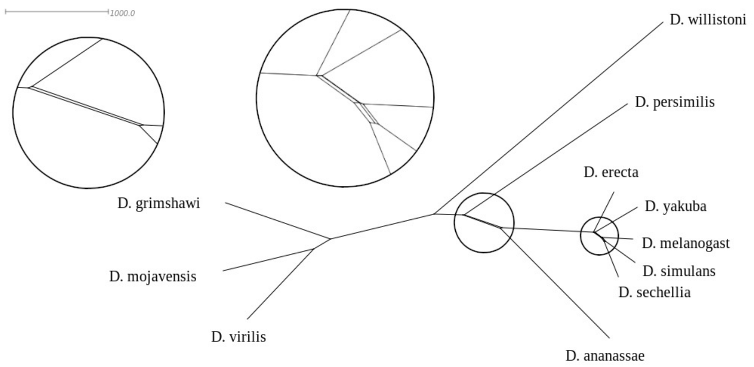

Fig. D4: Phylogenetic network generated from the distances in Table D6 by `SplitsTree4`. The fit calculated by `SplitsTree4` was 97.7%.

Calculating the deviation of the distances calculated by `ding-cf` and the tree path metric, we obtain an average of around 0.5% per entry with the largest relative difference being 2% for the distance of *D. melanogaster* and *D. simulans*.

Another way to analyse how well a distance matrix reflects a tree, is to perform a *split decomposition*. In short, a *split* is a generalized tree edge in the sense that it separates one subset of taxa from the rest. It is then possible to decompose the distance matrix into a subset of so-called d-splits and a residual matrix containing no further d-splits. The way d-splits are displayed by `SplitsTree` is as a set of parallel edges separating one set part of the graph from the other. Most importantly, if a split decomposition is applied to an *additive metric*, that is, the path metric of a unique tree, the only d-splits in the resulting network are the edges of that tree. For more information, the interested reader is referred to [15].

We used `SplitsTree4` to construct such a splits network and give the output in Figure D4. The resulting network is very tree-like in appearance with all major splits conforming to the tree computed for Figure 14. We provide zommed in versions of the minor splits displayed by `SplitsTree`. We see that relative to the "correct" splits with which they conflict, they have far smaller weight. In combination with the computed fit of 97.7% calculated by `SplitsTree` for this data, we can further confirm that the distances calculated by `ding-cf` are close to being additive on this dataset.

D.2 Additional Drosophila Diagrams

The matching diagrams for all pairs of the 11 analyzed drosophila can be found here: https://doi.org/10.6084/m9.figshare.24480892.v1
